# Supported Telemonitoring and Glycemic Control in People with Type 2 Diabetes: The Telescot Diabetes Pragmatic Multicenter Randomized Controlled Trial

**DOI:** 10.1371/journal.pmed.1002098

**Published:** 2016-07-26

**Authors:** Sarah H. Wild, Janet Hanley, Stephanie C. Lewis, John A. McKnight, Lucy B. McCloughan, Paul L. Padfield, Richard A. Parker, Mary Paterson, Hilary Pinnock, Aziz Sheikh, Brian McKinstry

**Affiliations:** 1 Usher Institute of Population Health Sciences and Informatics, University of Edinburgh, Edinburgh, Scotland, United Kingdom; 2 Edinburgh Napier University School of Nursing, Midwifery and Social Care, Edinburgh, Scotland, United Kingdom; 3 Metabolic Unit, Western General Hospital, Edinburgh, Scotland, United Kingdom; 4 College of Medicine and Veterinary Medicine, University of Edinburgh, Edinburgh, Scotland, United Kingdom; University of Cambridge, UNITED KINGDOM

## Abstract

**Background:**

Self-monitoring of blood glucose among people with type 2 diabetes not treated with insulin does not appear to be effective in improving glycemic control. We investigated whether health professional review of telemetrically transmitted self-monitored glucose results in improved glycemic control in people with poorly controlled type 2 diabetes.

**Methods and Findings:**

We performed a randomized, parallel, investigator-blind controlled trial with centralized randomization in family practices in four regions of the United Kingdom among 321 people with type 2 diabetes and glycated hemoglobin (HbA1c) >58 mmol/mol. The supported telemonitoring intervention involved self-measurement and transmission to a secure website of twice-weekly morning and evening glucose for review by family practice clinicians who were not blinded to allocation group. The control group received usual care, with at least annual review and more frequent reviews for people with poor glycemic or blood pressure control. HbA1c assessed at 9 mo was the primary outcome. Intention-to-treat analyses were performed. 160 people were randomized to the intervention group and 161 to the usual care group between June 6, 2011, and July 19, 2013. HbA1c data at follow-up were available for 146 people in the intervention group and 139 people in the control group. The mean (SD) HbA1c at follow-up was 63.0 (15.5) mmol/mol in the intervention group and 67.8 (14.7) mmol/mol in the usual care group. For primary analysis, adjusted mean HbA1c was 5.60 mmol/mol / 0.51% lower (95% CI 2.38 to 8.81 mmol/mol/ 95% CI 0.22% to 0.81%, *p* = 0·0007). For secondary analyses, adjusted mean ambulatory systolic blood pressure was 3.06 mmHg lower (95% CI 0.56–5.56 mmHg, *p* = 0.017) and mean ambulatory diastolic blood pressure was 2.17 mmHg lower (95% CI 0.62–3.72, *p* = 0.006) among people in the intervention group when compared with usual care after adjustment for baseline differences and minimization strata. No significant differences were identified between groups in weight, treatment pattern, adherence to medication, or quality of life in secondary analyses. There were few adverse events and these were equally distributed between the intervention and control groups. In secondary analysis, there was a greater number of telephone calls between practice nurses and patients in the intervention compared with control group (rate ratio 7.50 (95% CI 4.45–12.65, *p* < 0.0001) but no other significant differences between groups in use of health services were identified between groups. Key limitations include potential lack of representativeness of trial participants, inability to blind participants and health professionals, and uncertainty about the mechanism, the duration of the effect, and the optimal length of the intervention.

**Conclusions:**

Supported telemonitoring resulted in clinically important improvements in control of glycaemia in patients with type 2 diabetes in family practice. Current Controlled Trials, registration number ISRCTN71674628.

**Trial Registration:**

Current Controlled Trials ISRCTN 71674628

## Abstract 2

Sarah Wild and colleagues investigate whether a monitoring system that shares results with a patient's physician or nurse can help reduce HbA1c measurements for patients with type 2 diabetes.

## Author Summary

**Why Was This Study Done?:**

- Type 2 diabetes is a common condition for which self-management is very important.
- Telemonitoring is a system in which people with different conditions take measurements at home. The measurements are automatically shared with their doctor or nurse, who can give advice or change treatment.
- Self-monitoring of blood sugar levels by itself does not appear to be a good way of improving control of diabetes, and it is not known whether sharing blood sugar results with health professionals using telemonitoring improves diabetes control.

**What Did the Researchers Do and Find?:**

- We carried out a “randomized controlled trial” in which 321 people with type 2 diabetes, recruited from general practices across the United Kingdom, were split into two groups and studied over 9 mo.
- One group was looked after in the usual way. People in the telemonitoring group were asked to send measurements of their blood glucose, blood pressure, and weight from devices they were given. We asked practice nurses to check the results on the website at least once a week and to contact the patients by telephone or email to adjust treatment or reinforce lifestyle advice if necessary.
- We found that the people in the telemonitoring group had significantly better control of diabetes and blood pressure than the people who had treatment as usual.
- Telemonitoring made no significant difference in patients’ weight.

**What Do these Findings Mean?:**

- The study shows that telemonitoring can be effectively applied in people with type 2 diabetes in a research setting.
- Further work is required to identify whether telemonitoring is helpful in normal health care, which people find it most useful, and how long it should be used for.

## Introduction

The International Diabetes Federation has estimated that the number of people with diagnosed diabetes in the world in 2013 was 206 million [1]. Annual health care costs of a patient with type 2 diabetes are approximately two to three times higher than the costs of a person of similar age and sex without diabetes, and approximately 80% of the cost of diabetes to health services in developed countries is spent on complications. Control of blood glucose and blood pressure and management of dyslipidemia reduces complications and mortality among people with diabetes [2,3]. The increasing prevalence of diabetes and other chronic conditions means that traditional models of management in family practice are under extreme pressure and there is considerable interest in developing effective approaches to self-management across the world.

The traditional clinician-led model with regular face-to-face consultations for managing diabetes and hypertension is costly in terms of health care professionals’ time, rarely supports self-management by patients, and is often not very effective, partly because therapeutic inertia may result in reluctance to change treatments [4]. Systematic reviews indicate that engaging patients in self-monitoring and management can improve clinical outcomes in some chronic disorders (for example, asthma), but the evidence that self-monitoring of blood glucose is beneficial in people with type 2 diabetes is less clear [5,6]. This may be due to therapeutic inertia, poor adherence to both lifestyle advice and prescribed medication among people with diabetes [7,8], and the fact that patients become anxious when faced with self-monitored evidence of poor control when feedback from clinicians is infrequent [9].

The aim of the Telescot Diabetes Trial was to investigate the effect of supervised, self-monitoring of glycemic control, blood pressure, and weight with telemetric transmission of measurements (hereafter described as supported telemonitoring) among people with poorly controlled diabetes compared with a control group receiving usual care. The trial was performed within a program of similar trials in patients with chronic conditions described further at www.telescot.org and included a previous hypertension trial [10].

## Methods

### Study Design

We performed a randomized, parallel, investigator-blind, controlled trial in family practices in four regions of the United Kingdom (UK). The South-East Scotland Research Ethics Committee (reference number: 10/S1102160) approved the study, which was overseen by an Independent Trial Steering Committee. All participants provided written informed consent. The protocol provides details of the design and methods and is available as S1 Text and in the published version [11].

### Participants

We recruited family practices caring for socially diverse populations from Borders, Glasgow, and Lothian in Scotland and Kent in England through primary care research networks. Participating practices searched their registers of people with type 2 diabetes (assisted as necessary by researchers from Primary Care Research Networks) to identify and send an information sheet and letter inviting potential participants to respond to the research team if they were interested in participating in the trial.

Volunteers attended a screening visit at which the intervention and trial was explained, written informed consent was obtained, a blood sample for HbA1c and lipid measurement was collected and daytime ambulatory blood pressure monitoring (ABPM) was initiated. Inclusion criteria were the following: diagnosis of type 2 diabetes managed in family practice, age over 17 years, availability of a mobile telephone signal at home, and poor glycemic control, defined as HbA1c >58 mmol/mol. The original protocol included an inclusion criterion of poorly controlled blood pressure (based on average daytime self-monitored blood pressure ≥130 mmHg), but this criterion was removed and the protocol [11] amended in the first month of recruitment because the majority of potential participants had well-controlled blood pressure. After 15 months of recruitment the initial upper age limit was removed and we clarified that exclusion on the basis of surgery within the last 3 mo implied major surgery and that treated atrial fibrillation was not an exclusion criterion. The protocol changes mean that trial participants are more representative of the population with type 2 diabetes with poor glycemic control than if the inclusion requirements for poor blood pressure control and maximum age had remained.

Exclusion criteria were blood pressure >210/135 mmHg, hypertension or renal disease managed in secondary care, treatment for a cardiac event or other life-threatening illness within the previous 6 mo, major surgery within the last 3 mo, atrial fibrillation unless successfully treated or cardioverted, inability to use self-monitoring equipment, and pregnancy.

### Randomization and Masking

People meeting the eligibility criteria were randomized at a second visit using an allocated treatment code generated by a computer from a minimization procedure based on age (<70 or ≥70 years of age), sex, geographic area, prescription of two or more diabetes treatments, prescription of three or more anti-hypertensive medications, and whether they had previously monitored their blood sugars, as well as a 1:1 ratio for intervention to control group. Minimization was used to ensure that important baseline variables were balanced between intervention and control groups. To ensure unpredictability of the minimization procedure, there was a 1 in 10 chance that the determined treatment allocation was reversed; the corresponding random numbers list was stored securely at the Edinburgh Clinical Trials Unit and concealed from participants and research nurses. Random allocations were obtained by research nurses who enrolled participants through a secure web-based system prepared and maintained by the Edinburgh Clinical Trials Unit.

It was not possible to blind participants, clinicians, or research nurses to allocation group for all participants. In Lothian and Borders a different research nurse collected data at baseline and follow-up, although this was not possible for participants in Kent and Glasgow. The primary and principal secondary outcomes were based on objective measurements derived from collection of blood samples, ABPM, and weight measurement by research nurses such that lack of blinding is unlikely to introduce measurement bias. In addition, participants were asked not to reveal allocation until after final data collection was complete.

### Procedures

Research nurses undertook baseline measurements prior to randomization in all participants. Baseline measurements included HbA1c, the average daytime blood pressure (BP) from ABPM based on a minimum of 12 measurements recorded every 20 min between 7 a.m. and 11 p.m., average of second and third measurements of office BP measured by research nurses, smoking history, height and weight, exhaled carbon monoxide, questionnaire data on anxiety/depression (Hospital Anxiety and Depression Scale) [12], quality of life (EQ-5D) [13], self-efficacy [14], medication adherence [15], physical activity [16], knowledge of managing diabetes (based on responses from the first 14 items of the diabetes knowledge test) [17], and ethnic group based on categories included in the 2011 UK Census.

A full description of the intervention is provided in S3 Text. In brief, participants in the intervention group were given instructions for use of blood pressure, blood glucose, and weight monitors, that used Bluetooth technology to transmit readings via a supplied modem to a remote secure server by research nurses. The participant and their family practice professionals were able to access password-protected records on the server. Participants were asked to measure one fasting and one nonfasting blood glucose at least twice weekly and measure BP and weight at least weekly (with increased testing as recommended by the clinician for people treated with insulin). Participants were given advice on lifestyle modification, on lag time for effects of lifestyle and medication change on glucose and blood pressure, and when and how to contact their family practice team via research nurses. Primary care nurses were asked to check participants’ results weekly and to organize treatment changes based on national guidelines for diabetes and hypertension management (see protocol and S4 Text) [11]. The intervention lasted 9 mo, when patients were asked to attend for follow-up. The comparison group received usual care. Usual diabetes care in family practice is financially incentivized in the UK with targets set on a sliding scale of rewards for glycemic and blood pressure control (see [18] for more detail). Well-controlled patients are reviewed at least once a year, but more frequent reviews are performed for people who have poor glycemic or blood pressure control.

### Outcomes

The follow-up appointment included similar measurements and questionnaires to those performed at baseline. The primary outcome was adjusted mean difference in HbA1c, and secondary outcomes were adjusted mean differences in daytime ambulatory systolic and diastolic blood pressures and weight between treatment groups (see statistical analysis for further details of adjustments). HbA1c (that reflects blood glucose control over the previous 2–3 mo) recorded within 6 wk either side of the 9 mo follow-up point were used in order to maximize completeness of follow-up data. Standard blood pressure measures, serum total cholesterol, HDL cholesterol, urinary sodium/creatinine ratio, renal function (eGFR), United Kingdom Prospective Diabetes Study (UKPDS) risk score, anxiety and depression, quality of life, self-efficacy, self-reported physical activity, self-reported exercise tolerance, self-reported alcohol intake and diabetes knowledge, number of attendances at accident and emergency, at out-of-hours care, with practice nurse, with general practitioner (GP), hospital admissions, and number of telephone/email contacts with practice nurses and GPs were also compared between groups as pre-specified additional outcome measures. Use of health care resources (number and duration of hospital admissions, practice and out-of-hours consultations, routine reviews for BP or diabetes, prescriptions for antihypertensive and diabetes drugs) and adverse events were extracted from participants’ electronic family practice records by research nurses.

### Statistical Analysis

We used distribution of HbA1c and BP from the Lothian population-based diabetes register for 2008 (mean [SD] HbA1c of 68 [15] mmol/mol and mean [SD] systolic blood pressure of 149 [13] mmHg) when planning the trial (see protocol for further details). Using this information, we estimated that a randomized controlled trial with 125 people completing each arm with no change in values in the control group at the end of the trial would have 80% power at 5% significance to detect a 6 mmol/mol fall in HbA1c and a 5 mmHg fall in systolic BP in the intervention group. We aimed to recruit a total of 320 trial participants to allow for 20% attrition.

Data were analyzed on an intention-to-treat basis using a complete case analysis assuming missing outcome data were missing completely at random. Difference in mean HbA1c between intervention and control groups, the primary outcome, was estimated using linear regression with a 95% confidence interval for the difference, after adjusting for baseline HbA1c and with the addition of minimization strata (for which adjustment was accidentally omitted from the protocol) using analysis of covariance. Clustering by practice was investigated for the primary outcome by calculating the intra-cluster correlation coefficient. A priori planned sensitivity analyses involved (i) exclusion of participants with values of the primary outcome variable more than four standard deviations from the mean and (ii) a multiple imputation analysis using the same variables as in the main analysis (including the outcome variable) with the addition of weight, baseline ABPM systolic blood pressure, baseline ABPM diastolic blood pressure, baseline HDL cholesterol, and baseline total cholesterol, based on 30 imputation datasets, and combining results using Rubin’s rules [19]. Similar linear regression methods were used for systolic and diastolic BPs and weight and the other continuous secondary outcomes listed in the protocol. Negative binomial regression was used for outcomes measured as counts, adjusting for baseline and minimization strata, where possible. Logistic regression was used for binary outcomes, adjusting for the minimization strata. Subgroup analyses and tests for interactions were performed as described in the protocol and S5 Text [11]. Analyses were performed using version 9.3 of the SAS system for Windows (SAS Institute Inc., Cary, NC, United States). There was no data monitoring committee. The trial had the registration number ISRCTN71674628 in Current Controlled Trials.

## Results

**Fig 1:**
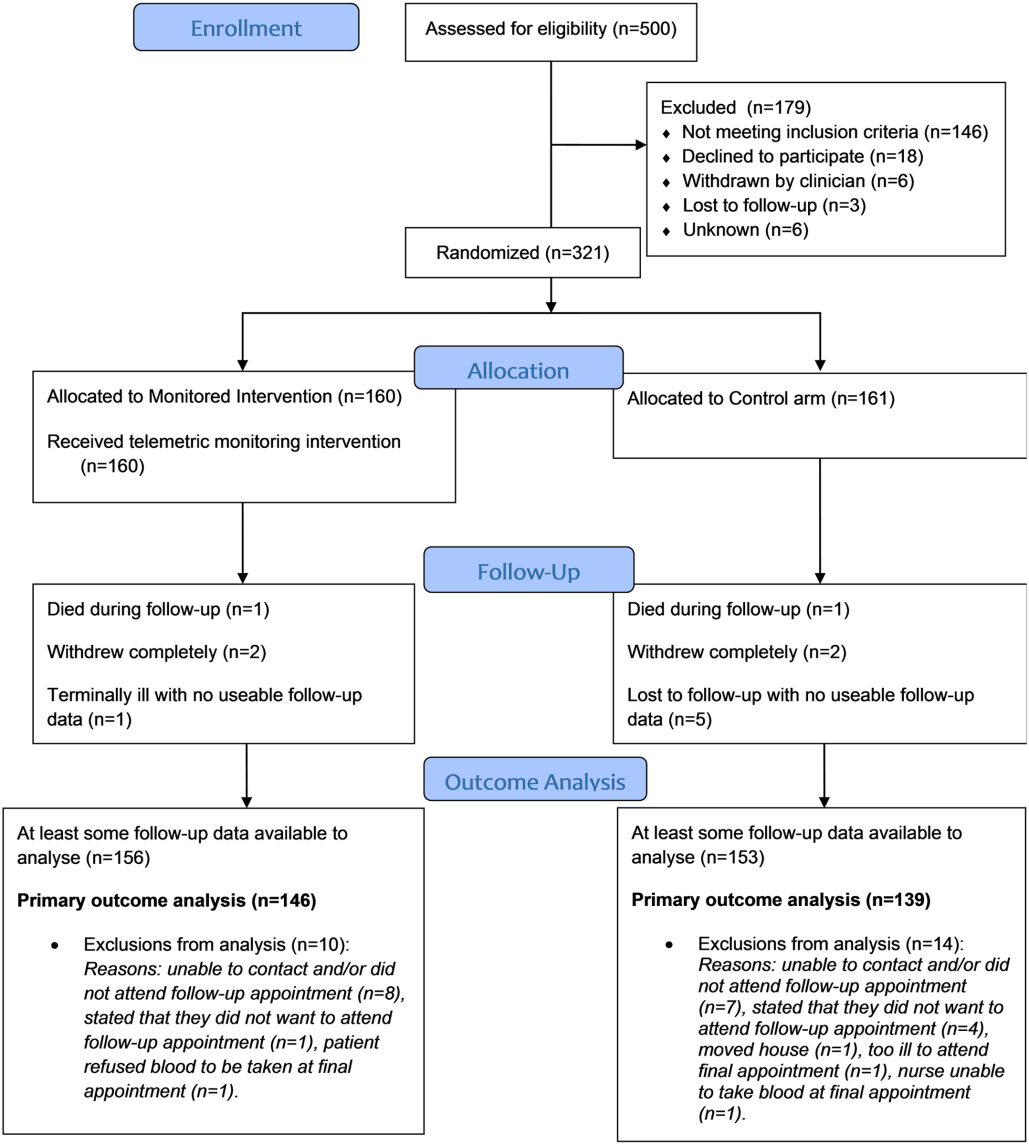
CONSORT flow chart for the Telescot diabetes trial.

Forty-four practices were invited to participate, of which 42 recruited participants (with a mean of 8 participants per practice, min 1, max 46). The intra-cluster correlation coefficient was calculated to be below zero (-0.0012) and so there was no evidence of clustering by practice. We invited 2,680 potential participants, 500 were assessed for eligibility, and 321 were randomized, 160 to the intervention and 161 to the control arms, respectively (see Fig 1: CONSORT flow chart). Just over half of the participants were recruited from Lothian and Borders. The first participant was randomized on June 6, 2011, and the last was randomized on July 19, 2013, with the last follow-up visit on May 21, 2014. Data for the primary outcome were available for 146 people (91%) in the intervention group and 139 people (86%) in the control group.

The two groups had similar distributions of relevant characteristics at baseline as shown in Tables 1 and 2 (summarizing distribution of minimization criteria for all randomized participants and participants contributing to the primary outcome analyses respectively) and Tables 3 and 4 (summarizing clinical characteristics for all randomized participants and participants contributing to the primary outcome analyses respectively). The mean (SD) age of participants was 61.0 (9.8) years with a range of 33.7 to 86.3 years; two-thirds were men and mean (SD) duration of diabetes was 7.4 (5.8) years. Twenty-five percent of participants in the intervention group submitted blood glucose readings as requested (one fasting and one nonfasting blood glucose at least twice weekly) with 21% of participants submitting less than a quarter of requested measurements.

**Table 1:** Distribution of minimization criteria in all trial participants by trial arm (supported telemonitoring compared to usual care).

	Trial arm			
	Supported telemonitoring		Usual care	
	*n*	%	*n*	%
**Total number of participants**	160	100.0	161	100.0
**Age group**	131	81.9	130	80.7
<70 years				
70+ years	29	18.1	31	19.3
**Sex**	106	66.3	108	67.1
Male				
Female	54	33.8	53	32.9
**Center**	88	55.0	89	55.3
Lothian				
Kent	61	38.1	61	37.9
Glasgow	10	6.3	8	5.0
Borders	1	0.6	3	1.9
**Diabetes drugs**	48	30.0	49	30.4
0–1				
2+	112	70.0	112	69.6
**Insulin users**	26	16.3	25	15.5
**Hypertension drugs**	132	82.5	129	80.1
0–2				
3+	28	17.5	32	19.9
**Glucose self-monitoring**	51	31.9	47	29.2
Never				
Occasionally	45	28.1	47	29.2
Regularly	64	40.0	67	41.6

**Table 2:** Distribution of minimization criteria in trial participants included in the primary endpoint analysis by trial arm (supported telemonitoring compared to usual care).

	Trial arm			
	Supported telemonitoring		Usual care	
	*n*	%	*n*	%
**Total number of participants**	146	100.0	139	100.0
**Age group**	119	81.5	112	80.6
<70 years				
70+ years	27	18.5	27	19.4
**Sex**	98	67.1	92	66.2
Male				
Female	48	32.9	47	33.8
**Center**	83	56.8	83	59.7
Lothian				
Kent	54	37.0	49	35.3
Glasgow	9	6.2	5	3.6
Borders	0	0.0	2	1.4
**Diabetes drugs**	43	29.5	42	30.2
0–1				
2+	103	70.5	97	69.8
**Insulin users**	22	15.1	17	12.2
**Hypertension drugs**	118	80.8	112	80.6
0–2				
3+	28	19.2	27	19.4
**Glucose self-monitoring**	48	32.9	44	31.7
Never				
Occasionally	40	27.4	36	25.9
Regularly	58	39.7	59	42.4

**Table 3:** Characteristics of all trial participants at baseline by trial arm (supported telemonitoring compared to usual care).

	Trial arm					
	Supported telemonitoring			Usual care		
	*n*	Mean	SD	*n*	Mean	SD
Age (years)	160	60.5	9.8	161	61.4	9.8
Duration of diabetes (years)	160	7.4	5.7	161	7.4	5.8
Height (cm)	160	170.7	9.8	161	170.9	8.9
Weight (kg)	160	98.8	23.2	161	93.1	19.8
BMI (kg/m2)	160	33.8	7.0	161	31.9	6.3
Alcohol units per week	160	6.5	13.8	161	5.6	8.8
Office based systolic blood pressure (mmHg)	160	139.4	14.6	160	137.6	13.6
Office based diastolic blood pressure (mmHg)	160	83.2	10.0	160	82.1	10.1
Average ambulatory daytime systolic blood pressure (mmHg)	157	134.0	11.9	153	134.9	11.6
Average ambulatory daytime diastolic blood pressure (mmHg)	157	78.9	8.8	153	78.6	8.7
HbA1c (%)	160	8.9	1.3	161	8.8	1.1
HbA1c (mmol/mol)	160	74.1	14.3	161	73.2	12.1

**Table 4:** Characteristics of trial participants included in the primary endpoint analysis at baseline by trial arm (supported telemonitoring compared to usual care).

	Trial arm					
	Supported telemonitoring			Usual care		
	*n*	Mean	SD	*n*	Mean	SD
Age (years)	146	60.9	9.4	139	61.9	9.3
Duration of diabetes (years)	146	7.5	5.8	139	7.2	5.8
Height (cm)	146	170.9	9.9	139	170.6	8.7
Weight (kg)	146	98.9	23.2	139	92.5	20.1
BMI (kg/m2)	146	33.8	7.0	139	31.8	6.4
Alcohol units per week	146	6.3	13.3	139	5.9	9.2
Office based systolic blood pressure (mmHg)	146	139.6	14.7	138	137.7	13.6
Office based diastolic blood pressure (mmHg)	146	82.8	9.9	138	81.8	10.3
Average ambulatory daytime systolic blood pressure (mmHg)	143	134.1	11.9	133	135.2	11.5
Average ambulatory daytime diastolic blood pressure (mmHg)	143	78.7	8.8	133	78.5	8.6
HbA1c (%)	146	8.9	1.3	139	8.8	1.1
HbA1c (mmol/mol)	146	74.1	14.5	139	73.0	11.7

Primary and secondary outcomes are described in detail in Table 5. The absolute mean difference in HbA1c between groups was 5.60 mmol/mol; (95% CI 2.38 to 8.81 mmol/mol; *p* = 0.0007) lower in the monitored compared with the control arm. The effect size was similar after removing outliers more than four standard deviations away from the mean in the primary outcome variable in a sensitivity analysis: 5.32 mmol/mol 95% CI 2.12 to 8.52 mmol/mol, *p* = 0.0012. After performing multiple imputation, the difference was estimated to be 4.75 95% CI 1.55 to 7.95 mmol/mol, *p* = 0.0036.

**Table 5:** Baseline and follow-up values for primary and secondary outcomes in the trial by trial arm.

Outcome Variable	*n* *	Baseline		Follow-up		Adjusted Mean difference** (Supported tele monitoring–Usual care)	95% Confidence Limits for adjusted mean difference		*p*
		Supported tele-monitoring Mean (SD)	Usual care Mean (SD)	Supported tele-monitoring Mean (SD)	Usual care Mean (SD)				
HbA1c (mmol/mol)	285 (146:139)	74.1	73.0 (11.7)	63.0 (15.6)	67.8 (14.7)	-5.60	-8.81	-2.38	0.007
HbA1c (%)	285 (146:139)	8.9 (1.3)	8.8 (1.1)	7.9 (1.4)	8.4 (1.3)	-0.51	-0.81	-0.22	0.007
Average ambulatory daytime systolic blood pressure (mmHg)	229 (121:108)	133.7 (11.3)	133.8 (10.5)	131.0 (11.9)	133.8 (11.3)	-3.06	-5.56	-0.56	0.0166
Average ambulatory daytime diastolic blood pressure (mmHg)	229 (121:108)	78.5 (8.4)	77.9 (8.5)	76.2 (8.8)	77.7 (8.5)	-2.17	-3.72	-0.62	0.0063
Weight (kg)	280 (145:135)	98.8 (23.0)	92.7 (20.2)	96.9 (22.0)	91.5 (20.5)	-0.35	-1.54	0.83	0.557

*Sample size per group is shown in brackets (Supported telemonitoring:Usual Care)

**Adjusted for baseline, age over 70 years, sex, center, number of diabetes drugs, number of anti-hypertension drugs, and frequency of glucose self-monitoring.

Ambulatory BP data were available at follow-up for 229 people, and BP was lower in the intervention than control group: systolic BP by 3.06 mmHg; 95% CI 0.56 to 5.56 mm Hg; *p* = 0.017 and diastolic BP by 2.17 mmHg; 95% CI 0.62 to 3.72 mmHg; *p* = 0.006. Weight at follow-up was not significantly different between trial arms (0.35 kg lower for the 145 people in the intervention compared to the 135 people in control group for whom data were available; 95% CI -0.83 to 1.54 kg, *p* = 0.557).

Additional outcome measures are reported in S1 and S2 Tables. Prescribing patterns for diabetes treatments or anti-hypertensive drugs were similar between intervention and control groups as demonstrated in Tables 6 and 7. Insulin treatment was started during follow-up for eight people in the intervention group and six people in the control group. In addition, post-hoc linear regression models adjusted for baseline defined daily doses (DDDs) [20] and minimization variables did not identify statistically significant differences in mean DDD at follow-up of either diabetes treatment (0.003 [95% CI -0.21 to 0.21]) or anti-hypertensive treatment (0.18 [95% CI -0.04 to 0.39]) between groups. In particular, there was no evidence that the intervention increased anxiety levels. The only statistically significant difference between randomization groups in use of health services was a greater number of telephone calls between practice nurses and patients in the intervention compared with control group (rate ratio 7.50 [95% CI 4.45 to 12.65, *p* < 0.0001]) (S3 Table). The intervention cost significantly more than usual care (mean difference per patient £286.00 [95% CI £154.27 to £409.62]) due to telemonitoring service costs and additional nurse phone consultations. Health economic modelling of the cost-effectiveness of the intervention is in progress and will be reported separately. There were few adverse events that could be attributed to diabetes or BP control and these were equally distributed between the intervention and control groups (S4 Table provides further detail).

**Table 6:** Distribution of differences in numbers of diabetes treatments prescribed between baseline and follow-up in Telescot diabetes trial participants by trial arm and initiation of insulin during follow-up.

Difference in number of diabetes medications	Patient group	Allocated treatment		Total
		Telemetric monitoring	Usual care	
**-2**	All	1 (1%)	0	1 (0.3%)
	Converted to Insulin	0	0	0
**-1**	All	8 (5%)	6 (4%)	14 (5%)
	Converted to Insulin	3 (2%)	1 (1%)	4 (1%)
**0**	All	113 (73%)	112 (74%)	225 (73%)
	Converted to Insulin	0	4 (3%)	4 (1%)
**1**	All	31 (20%)	32 (21%)	63 (21%)
	Converted to Insulin	4 (3%)	4 (3%)	8 (3%)
**2**	All	2 (1%)	2 (1%)	4 (1%)
	Converted to Insulin	1 (1%)	1 (1%)	2 (1%)
**Total**		155	152	307

**Table 7:** Distribution of differences in numbers of anti-hypertensive medications prescribed between baseline and follow-up in Telescot diabetes trial participants by trial arm and initiation of insulin during follow-up.

Difference in number of anti-hypertensive medications	Patient group	Allocated treatment		Total
		Telemetric monitoring	Usual care	
**-2**	All	0	1 (1%)	1 (0.4%)
	Converted to Insulin	0	0	0
**-1**	All	4 (3%)	3 (3%)	7 (3%)
	Converted to Insulin	0	0	0
**0**	All	89 (75%)	92 (80%)	181 (77%)
	Converted to Insulin	5 (4%)	6 (5%)	11 (5%)
**1**	All	19 (16%)	16 (14%)	35 (15%)
	Converted to Insulin	2 (2%)	2 (2%)	4 (2%)
**2**	All	7 (6%)	3 (3%)	10 (4%)
	Converted to Insulin	0	0	0
**Total**		119	115	234

There were no statistically significant interactions with group allocation for any of the pre-specified sub-groups of age, sex, socioeconomic status, baseline HbA1c, monitored systolic blood pressure, or body mass index (see S5 Text and S5–S10 Tables).

## Discussion

In this multicenter pragmatic trial based in UK family practice we observed clinically and statistically significantly greater improvements in glycemic control among people with poor glycemic control of type 2 diabetes who were offered supported telemonitoring over 9 mo than among the comparison group offered usual care. There were also significant reductions in blood pressure. The key feature of our trial is robust evidence suggesting that blood glucose monitoring with relatively little additional support from health professionals can be of value in terms of improving glycemic control in people who have previously had poor glycemic control. This finding contrasts with that of a systematic review published previously that suggested that unsupported self-monitoring of blood glucose does not have clinically significant beneficial effects [21].

The effect size (5·5 mmol/mol) we observed in our trial is the same as that used to establish efficacy of new drugs by the National Institute for Health and Care Excellence (NICE) algorithms and guidelines [22]. Similar improvements in systolic and diastolic BP to those observed in this trial of 3.3 and 1.4 mmHg were reported in the Heart Outcome Prevention Evaluation (HOPE) study and were associated with a 22% reduction in relative risk of cardiovascular mortality, myocardial infarction, or stroke [23]. In contrast to trials of strict glycemic control, mean weight did not increase in the intervention group. The lack of change in weight may reflect dietary changes or may be related to shorter follow-up in this trial compared to trials of intensive glycemic control [24]. In contrast to many other non-pharmacological interventions, this was achieved with a relatively small increase in researcher or health professional workload.

The strengths of this trial include recruitment from family practice of people with type 2 diabetes who have poor glycemic control despite the incentives offered to family practitioners for controlling diabetes and who constitute a more representative group than people participating in trials at specialist centers. In addition, diabetes management continued to take place in normal family practice rather than in a research setting. Objective measures were used for primary and principal secondary outcomes of glycemic control (HbA1c), BP (ABPM) and weight. There was limited loss to follow-up, and the sensitivity analyses and tests for interaction suggest that the reported findings are robust. However, the nature of trials is that although the majority of family practices who were invited agreed to participate, only 12% of people identified as potentially eligible from this challenging group of patients were randomized in this trial. It is not possible to determine what proportion of the general population of people with type 2 diabetes would wish to use telemonitoring if it were provided as a service and whether they would all be able to achieve similar results. Further work is required to determine what barriers there may be to implement telehealth in this group at scale, given the large numbers of people with poorly controlled type 2 diabetes (almost 100,000 people, or 2% of the population in Scotland alone). Additionally it is important to identify whether a shorter intervention may be equally clinically effective (and potentially more cost-effective than a longer intervention) or whether ongoing monitoring is required to maintain the effect.

The results of previous similar studies are conflicting, which may reflect the quality of the research, variation in settings between research clinics and normal clinical practice and the diverse nature of complex interventions. In a systematic review and meta-analysis of data from 5,069 patients participating in 26 studies of home telehealth for diabetes, the weighted mean difference in HbA1c between home telemonitoring and comparison groups was -0.21% (95% CI -0.35 to -0.08) in the available 21 studies, which were noted to be of variable methodological quality [25]. A meta-analysis of data from 2,552 participants in six clinical trials of self-monitoring of blood glucose among people with non-insulin treated type 2 diabetes reported a mean reduction in HbA1c of 2.7 (95% CI 1.6 to 3.9) mmol/mol at 6 mo in the intervention compared with control group, which was consistent across all strata and was described as a statistically significant, but not clinically meaningful result [21]. The results of the type 2 diabetes component of the Whole Systems Demonstrator cluster randomized controlled trial with 12 mo follow-up were similar in that HbA1c was lower by 0.21% or 2.3 mmol/mol (95% CI, 0.04% to 0.38, *p* = 0.013) in the intervention [26]. However a meta-analysis of 13 randomized controlled trials of telemedicine among 4,207 people with diabetes reported a statistically significant and clinically relevant absolute decline in HbA1c level in the intervention compared to control groups similar to our finding (mean difference -4.8 mmol/mol and 95% CI 6.7 to -2.8 mmol/mol; *p* < 0.001), but with evidence of heterogeneity between trials [27].

The International Diabetes Federation has identified seven essential elements of self-monitoring of blood glucose (SMBG): patient education, provider education, structured SMBG profile, SMBG goals, feedback, data used to modify treatment, and interactive communication or shared decision making [28]. Our intervention included all of these elements and, as for all complex interventions, it is not clear which component had the most effect and what effect telemonitoring had relative to more frequent contacts (e.g., office visits and telephone calls) or better tailored advice. A recent systematic review found that none of 15 studies of telemonitoring interventions in type 2 diabetes, all of which were performed outside the UK, included all of these elements, but those that included five or more achieved significant improvements in HbA1c [29].

A further meta-analysis of 23 trials with 7,037 participants and high levels of heterogeneity reported that home BP telemonitoring improved office systolic blood pressure by 4.71 (95% CI 3.24 to 6.18) and diastolic blood pressure by 2.45 (1.57 to 3.33) mmHg [30]. One of the meta-analyses mentioned above also summarized the effect of telemedicine on systolic BP from eight trials performed among people with diabetes, reporting no significant effect (-1.6 mmHg; 95% CI -7.2 to 4.1 mmHg; *p* = 0.585) [27]. It is important to note that these trials were performed in people with poorly controlled blood pressure, whereas our trial participants on average had reasonable blood pressure control at baseline and that we used the gold standard of ambulatory blood pressure monitoring as the outcome.

We were surprised that prescribing patterns did not differ markedly between the two arms of trial participants as our, and other, blood pressure telemonitoring studies reported increased anti-hypertensive prescribing in the intervention arm [10,30]. Improved glycemic control is frequently associated with increased weight, possibly due to failure to lose calories through glycosuria [24], but we observed no difference in weight change by trial arm. These findings suggest that the effect may have been mediated by improved self-management, resulting in changes in diet and lifestyle or increased adherence to drug treatment, rather than intensification of treatment. This explanation is supported by the findings of the nested qualitative study performed within the trial, in which participants reported that both the information they received from self-monitoring and knowledge that their glycemic control was being observed encouraged them to make lifestyle changes [31]. Unpublished data from the qualitative research suggest that reasons for not submitting glucose measurements included technical problems from the start and forgetting to take readings, particularly when away from home. In contrast, the responses to relevant questionnaires did not provide any evidence of improved concordance with lifestyle recommendations or treatment, but it is possible that the questionnaires may not be sensitive to such changes, as has been suggested by other studies of the performance of some of the questionnaires [32,33].

In summary, telemonitoring and supported self-management of blood glucose can result in clinically meaningful improvements in blood glucose among people with poorly controlled type 2 diabetes managed in routine family practice while requiring relatively small increases in clinician workload. There are also additional benefits for control of blood pressure in this population. Further research is required to determine if these changes are sustained over time and can be achieved if the intervention is used more widely or when targeted at specific patient groups.

## Supporting Information

S1 Table Further categorical secondary outcomes. (DOCX)

S2 Table Further continuous secondary outcomes. (DOCX)

S3 Table Differences in health service use. (DOCX)

S4 Table Adverse events occurring during the trial by randomization group. (DOCX)

S5 Table Results of subgroup analysis by tertiles of age. (DOCX)

S6 Table Results of subgroup analysis by sex. (DOCX)

S7 Table Results of subgroup analysis by tertiles of socioeconomic status. (DOCX)

S8 Table Results of subgroup analysis by tertiles of HbA1c. (DOCX)

S9 Table Results of subgroup analysis by tertiles of monitored systolic blood pressure. (DOCX)

S10 Table Results of subgroup analysis by tertiles of body mass index. (DOCX)

S1 Text Protocol. (PDF)

S2 Text CONSORT checklist. (DOC)

S3 Text Supplementary information about the intervention. (DOCX)

S4 Text Treatment algorithms for blood pressure and diabetes. (DOCX)

S5 Text Supplementary information about the subgroup analysis. (DOCX)

## Abbreviations

ABPM: ambulatory blood pressure monitoring
BP: blood pressure
DDDs: defined daily doses
GP: general practitioner
HOPE: Heart Outcome Prevention Evaluation
NICE: National Institute for Health and Care Excellence
SMBG: self-monitoring of blood glucose
UKPDS: United Kingdom Prospective Diabetes Study
