# Correction: Supported Telemonitoring and Glycemic Control in People with Type 2 Diabetes: The Telescot Diabetes Pragmatic Multicenter Randomized Controlled Trial

**DOI:** 10.1371/journal.pmed.1002163

**Published:** 2016-10-19

**Authors:** Sarah H. Wild, Janet Hanley, Stephanie C. Lewis, John A. McKnight, Lucy B. McCloughan, Paul L. Padfield, Richard A. Parker, Mary Paterson, Hilary Pinnock, Aziz Sheikh, Brian McKinstry

S3 Table and S4 Table files each contained the S3 Table. The inclusion of S4 Table can be viewed below.

## Supporting Information

S4 Table Adverse events occurring during the trial by randomization group. This file includes supplementary data.
(DOCX)
