# Supplementary material for: Supported Telemonitoring and Glycemic Control in People with Type 2 Diabetes: The Telescot Diabetes Pragmatic Multicenter Randomized Controlled Trial
Source: PLoS Med. 2016 Jul 26;13(7):e1002098. doi: 10.1371/journal.pmed.1002098 (PMC4961438; doi:10.1371/journal.pmed.1002098)
Supplement: S1 Table — (DOCX) [file pmed.1002098.s001.docx]

**S1 Table Further categorical secondary outcomes in the Telescot diabetes trial identified from multiple logistic regression analyses adjusting for the minimisation variables, odds ratios comparing monitored and not monitored participants**

| ***Outcome Variable*** | ***N**** | ***Number (%) - Monitored arm*** | ***Number (%) - Not monitored arm*** | ***Adjusted Odds Ratio*** | ***95% Confidence Limits of Odds Ratio*** | | ***Adjusted***  ***Risk Difference*** | ***95% Confidence Limits of Risk Difference*** | |
| --- | --- | --- | --- | --- | --- | --- | --- | --- | --- |
| Increased number of medications during follow-up | 308  (156:152) | 53 (34%) | 47 (31%) | 1.184 | 0.716 | 1.958 | 0.037 | -0.067 | 0.158 |
| Alcohol | 286  (148:138) | 73 (49%) | 72 (52%) | 0.877 | 0.541 | 1.422 | -0.033 | -0.151 | 0.086 |
| Current smoker | 288  (148:140) | 29 (20%) | 21 (15%) | 1.388 | 0.740 | 2.603 | 0.047 | -0.034 | 0.165 |
| Forgetting to take medication | 272  (140:132) | 63 (45%) | 61 (46%) | 0.967 | 0.594 | 1.575 | -0.008 | -0.124 | 0.113 |
| Careless taking medication | 272  (140:132) | 22 (16%) | 27 (20%) | 0.740 | 0.391 | 1.402 | -0.045 | -0.113 | 0.060 |

*Sample size per group is shown in brackets (Supported telemonitoring : Usual Care)
