# Supplementary material for: Supported Telemonitoring and Glycemic Control in People with Type 2 Diabetes: The Telescot Diabetes Pragmatic Multicenter Randomized Controlled Trial
Source: PLoS Med. 2016 Jul 26;13(7):e1002098. doi: 10.1371/journal.pmed.1002098 (PMC4961438; doi:10.1371/journal.pmed.1002098)
Supplement: S2 Table — (DOCX) [file pmed.1002098.s002.docx]

**S2 Table Further continuous secondary outcomes in the Telescot diabetes trial identified from multiple linear regression analyses adjusting for baseline values and variables used for minimisation.**

| **Outcome Variable** | **N*** | **Baseline** | | **Follow-up** | | **Adjusted Mean difference (Monitored – Not Monitored)** | **95% Confidence Limits for adjusted mean difference** | |
| --- | --- | --- | --- | --- | --- | --- | --- | --- |
|  |  | **Monitored**  **Mean (SD)** | **Not Monitored Mean (SD)** | **Monitored**  **Mean (SD)** | **Not Monitored Mean (SD)** |  |  |  |
| Office-based systolic blood pressure (mmHg) | 276 (143:133) | 139.4 (14.8) | 137.7 (13.8) | 134.2 (14.3) | 136.2 (14.7) | -2.31 | -5.41 | 0.79 |
| Office-based diastolic blood pressure (mmHg) | 276  (143:133) | 82.7 (9.7) | 81.9 (10.3) | 79.0 (9.6) | 80.6 (10.2) | -2.05 | -3.94 | -0.16 |
| HDL cholesterol (mmol/l) | 262  (141:121) | 1.1 (0.4) | 1.1 (0.2) | 1.1 (0.2) | 1.1 (0.3) | -0.04 | -0.08 | 0.01 |
| Total cholesterol(mmol/l) | 278  (145:133) | 4.2 (1.0) | 4.3 (1.0) | 4.1 (0.9) | 4.3 (1.2) | -0.15 | -0.33 | 0.02 |
| Urinary sodium/creatinine ratio | 203  (109:94) | 12.4 (6.4) | 12.8 (6.5) | 12.8 (8.7) | 12.8 (8.2) | 0.45 | -1.79 | 2.68 |
| Alcohol units per week | 286  (148:138) | 6.1 (13.2) | 5.9 (9.2) | 5.9 (14.8) | 5.3 (8.4) | 0.50 | -1.36 | 2.37 |
| CHD risk score at 10 years (UKPDS risk score %) | 249  (137:112) | 23.7 (13.4) | 25.7 (14.8) | 21.2 (12.5) | 25.2 (15.3) | -2.44 | -4.18 | -0.70 |
| Total HADS score | 257  (130:127) | 9.4 (7.4) | 9.1 (6.5) | 9.5 (7.8) | 9.6 (7.6) | -0.31 | -1.39 | 0.78 |
| EQ-5D index | 266  (136:130) | 0.8 (0.3) | 0.8 (0.2) | 0.8 (0.3) | 0.8 (0.3) | 0.00 | -0.04 | 0.05 |
| Self-efficacy | 265  (135:130) | 46.4 (11.7) | 47.1 (10.8) | 45.9 (12.9) | 45.9 (11.7) | 0.69 | -1.28 | 2.66 |
| Medication adherence | 270  (139:131) | 0.7 (0.9) | 1.0 (1.0) | 0.7 (0.9) | 0.8 (1.0) | 0.02 | -0.17 | 0.20 |
| Self-reported total physical activity score (IPAQ) | 241 (123:118) | 2209 (2785) | 2729 (3070) | 2323 (2772) | 3022 (3338) | -467.31 | -1142.88 | 208.27 |
| Diabetes Knowledge (first 14 items only) | 269 (138:131) | 8.9 (2.6) | 8.8 (2.0) | 9.0 (2.5) | 9.0 (2.3) | 0.04 | -0.42 | 0.49 |

*Sample size per group is shown in brackets (Supported telemonitoring : Usual Care)
