# Supplementary material for: Supported Telemonitoring and Glycemic Control in People with Type 2 Diabetes: The Telescot Diabetes Pragmatic Multicenter Randomized Controlled Trial
Source: PLoS Med. 2016 Jul 26;13(7):e1002098. doi: 10.1371/journal.pmed.1002098 (PMC4961438; doi:10.1371/journal.pmed.1002098)
Supplement: S4 Table — (DOCX) [file pmed.1002098.s004.docx]

**S3 Table Differences in health service use between randomization groups estimated from negative binomial regression models on the attendance and phone call count data adjusted for minimisation stratifiers** (*Note: Problem with model fitting due to a particularly small number of events in model 4) *Sample size per group is shown in brackets (Supported telemonitoring : Usual Care)

| **Model** | **Outcome Variable** | **N*** | **Average estimated yearly count - Monitored arm (SD)** | **Average estimated yearly count - Not monitored arm (SD)** | **Adjusted Rate Ratio** | **95% Confidence Limits of Rate Ratio** | |
| --- | --- | --- | --- | --- | --- | --- | --- |
| 1 | GP attendance count | 307 (156:151) | 5.0 (4.3) | 4.7 (3.7) | 1.00 | 0.85 | 1.19 |
|  |  |  |  |  |  |  |  |
| 2 | Practice Nurse  attendance count | 307 (156:151) | 5.2 (6.1) | 4.3 (5.0) | 1.18 | 0.97 | 1.43 |
|  |  |  |  |  |  |  |  |
| 3 | A&E attendance count | 307 (156:151) | 0.2 (0.6) | 0.2 (0.5) | 1.18 | 0.64 | 2.19 |
|  |  |  |  |  |  |  |  |
| 4 | Out-of-hours  attendance count* | 307 (156:151) | 0.1 (0.3) | 0.1 (0.3) | 0.82 | 0.33 | 2.05 |
|  |  |  |  |  |  |  |  |
| 5 | Hospital  attendance count | 307 (156:151) | 0.7 (1.7) | 0.8 (1.7) | 0.84 | 0.53 | 1.34 |
|  |  |  |  |  |  |  |  |
| 6 | Email contact count | 306 (156:150) | 0.0 (0.1) | 0.0 (0.2) | 0.26 | 0.02 | 3.67 |
|  |  |  |  |  |  |  |  |
| 7 | GP Phone count | 306 (156:150) | 1.4 (3.0) | 1.1 (1.8) | 1.06 | 0.68 | 1.63 |
|  |  |  |  |  |  |  |  |
| 8 | Practice Nurse  phone count | 307 (156:151) | 2.9 (4.9) | 0.3 (1.4) | 7.50 | 4.45 | 12.65 |
|  |  |  |  |  |  |  |  |
|  |  |  |  |  |  |  |  |
