# Supplementary material for: Supported Telemonitoring and Glycemic Control in People with Type 2 Diabetes: The Telescot Diabetes Pragmatic Multicenter Randomized Controlled Trial
Source: PLoS Med. 2016 Jul 26;13(7):e1002098. doi: 10.1371/journal.pmed.1002098 (PMC4961438; doi:10.1371/journal.pmed.1002098)
Supplement: S5 Table — (DOCX) [file pmed.1002098.s005.docx]

**S5 table: results of sub-group analysis by tertiles of age for the Telescot diabetes pragmatic randomized controlled trial**

| *Parameter Estimates - Age* | | | | | | | |
| --- | --- | --- | --- | --- | --- | --- | --- |
| *Variable* | *DF* | *Parameter Estimate* | *Standard Error* | *t Value* | *Pr > \|t\|* | *95% Confidence Limits* | |
| *Intercept* | 1 | 43.79597 | 5.70779 | 7.67 | <.0001 | 32.55854 | 55.03341 |
| *Supported telemonitoring* | 1 | -8.39788 | 3.00283 | -2.80 | 0.0055 | -14.30981 | -2.48595 |
| *Subgroup 1* | 1 | -4.10526 | 2.96076 | -1.39 | 0.1667 | -9.93436 | 1.72384 |
| *Subgroup 2* | 1 | -1.56539 | 3.06957 | -0.51 | 0.6105 | -7.60873 | 4.47795 |
| *Interaction 1* | 1 | 5.43313 | 4.08224 | 1.33 | 0.1843 | -2.60394 | 13.47019 |
| *Interaction 2* | 1 | 2.23730 | 4.13472 | 0.54 | 0.5889 | -5.90309 | 10.37769 |
| *Baseline HbA1c* | 1 | 0.39902 | 0.06454 | 6.18 | <.0001 | 0.27196 | 0.52609 |
| *Female sex* | 1 | -0.09267 | 1.76849 | -0.05 | 0.9582 | -3.57445 | 3.38911 |
| *Centre: Lothian* | 1 | -1.94411 | 1.83236 | -1.06 | 0.2896 | -5.55165 | 1.66342 |
| *Centre: Glasgow* | 1 | 4.27747 | 4.06773 | 1.05 | 0.2939 | -3.73104 | 12.28598 |
| *Centre: Borders* | 1 | -12.06019 | 9.99670 | -1.21 | 0.2287 | -31.74159 | 7.62120 |
| *Two or more Diabetes Drugs* | 1 | -5.64014 | 1.95093 | -2.89 | 0.0042 | -9.48111 | -1.79917 |
| *Three or more Anti-hypertension Drugs* | 1 | -3.54920 | 2.14651 | -1.65 | 0.0994 | -7.77522 | 0.67683 |
| *Never used glucose monitoring* | 1 | -0.03735 | 2.04716 | -0.02 | 0.9855 | -4.06778 | 3.99308 |
| *Occasional glucose monitoring* | 1 | 2.36071 | 2.06975 | 1.14 | 0.2551 | -1.71419 | 6.43561 |
