# Supplementary material for: Supported Telemonitoring and Glycemic Control in People with Type 2 Diabetes: The Telescot Diabetes Pragmatic Multicenter Randomized Controlled Trial
Source: PLoS Med. 2016 Jul 26;13(7):e1002098. doi: 10.1371/journal.pmed.1002098 (PMC4961438; doi:10.1371/journal.pmed.1002098)
Supplement: S6 Table — (DOCX) [file pmed.1002098.s006.docx]

**S6 table: results of sub-group analysis by sex for the Telescot diabetes pragmatic randomized controlled trial**

| *Parameter Estimates - Sex* | | | | | | | |
| --- | --- | --- | --- | --- | --- | --- | --- |
| *Variable* | *DF* | *Parameter Estimate* | *Standard Error* | *t Value* | *Pr > \|t\|* | *95% Confidence Limits* | |
| *Intercept* | 1 | 40.11722 | 5.26025 | 7.63 | <.0001 | 29.76125 | 50.47319 |
| *Supported telemonitoring* | 1 | -5.57824 | 2.00980 | -2.78 | 0.0059 | -9.53498 | -1.62149 |
| *Female* | 1 | -0.24235 | 2.48104 | -0.10 | 0.9223 | -5.12683 | 4.64213 |
| *Gender Interaction* | 1 | -0.05812 | 3.48868 | -0.02 | 0.9867 | -6.92637 | 6.81013 |
| *Baseline HbA1c* | 1 | 0.40761 | 0.06339 | 6.43 | <.0001 | 0.28281 | 0.53240 |
| *Over 70 years old* | 1 | 3.18312 | 2.12969 | 1.49 | 0.1362 | -1.00965 | 7.37588 |
| *Centre: Lothian* | 1 | -1.54718 | 1.80076 | -0.86 | 0.3910 | -5.09238 | 1.99802 |
| *Centre: Glasgow* | 1 | 4.43840 | 3.98306 | 1.11 | 0.2661 | -3.40314 | 12.27995 |
| *Centre: Borders* | 1 | -11.02443 | 9.93362 | -1.11 | 0.2681 | -30.58098 | 8.53212 |
| *Two or more Diabetes Drugs* | 1 | -5.36635 | 1.91959 | -2.80 | 0.0055 | -9.14550 | -1.58720 |
| *Three or more Anti-hypertension Drugs* | 1 | -3.58572 | 2.11762 | -1.69 | 0.0915 | -7.75473 | 0.58329 |
| *Never used glucose monitoring* | 1 | 0.01040 | 2.04344 | 0.01 | 0.9959 | -4.01257 | 4.03337 |
| *Occasional glucose monitoring* | 1 | 2.47362 | 2.04896 | 1.21 | 0.2284 | -1.56021 | 6.50745 |
