# Supplementary material for: Supported Telemonitoring and Glycemic Control in People with Type 2 Diabetes: The Telescot Diabetes Pragmatic Multicenter Randomized Controlled Trial
Source: PLoS Med. 2016 Jul 26;13(7):e1002098. doi: 10.1371/journal.pmed.1002098 (PMC4961438; doi:10.1371/journal.pmed.1002098)
Supplement: S7 Table — (DOCX) [file pmed.1002098.s007.docx]

**S7 table: results of sub-group analysis for tertile of Scottish Index of Multiple Deprivation (SIMD) for Scottish participants in the Telescot diabetes pragmatic randomized controlled trial**

| *Parameter Estimates – Tertile SIMD* | | | | | | | |
| --- | --- | --- | --- | --- | --- | --- | --- |
| *Variable* | *DF* | *Parameter Estimate* | *Standard Error* | *t Value* | *Pr > \|t\|* | *95% Confidence Limits* | |
| *Intercept* | 1 | 40.21653 | 5.26193 | 7.64 | <.0001 | 29.85672 | 50.57633 |
| *Supported telemonitoring* | 1 | -6.63232 | 2.12328 | -3.12 | 0.0020 | -10.81268 | -2.45197 |
| *Subgroup 1* | 1 | -4.17028 | 3.35109 | -1.24 | 0.2144 | -10.76799 | 2.42742 |
| *Subgroup 2* | 1 | -1.15498 | 3.27504 | -0.35 | 0.7246 | -7.60295 | 5.29300 |
| *Interaction 1* | 1 | 2.16447 | 4.25609 | 0.51 | 0.6115 | -6.21501 | 10.54396 |
| *Interaction 2* | 1 | 3.16556 | 4.26123 | 0.74 | 0.4582 | -5.22404 | 11.55516 |
| *Baseline HbA1c* | 1 | 0.41179 | 0.06351 | 6.48 | <.0001 | 0.28674 | 0.53683 |
| *Over 70 years old* | 1 | 3.44605 | 2.15793 | 1.60 | 0.1115 | -0.80253 | 7.69463 |
| *Female sex* | 1 | -0.19148 | 1.75978 | -0.11 | 0.9134 | -3.65617 | 3.27321 |
| *Centre: Lothian* | 1 | -0.71950 | 2.26518 | -0.32 | 0.7510 | -5.17923 | 3.74024 |
| *Centre: Glasgow* | 1 | 5.49427 | 4.17883 | 1.31 | 0.1897 | -2.73311 | 13.72164 |
| *Centre: Borders* | 1 | -7.32920 | 10.35348 | -0.71 | 0.4796 | -27.71337 | 13.05497 |
| *Two or more Diabetes Drugs* | 1 | -5.48377 | 1.93599 | -2.83 | 0.0050 | -9.29539 | -1.67216 |
| *Three or more Anti-hypertension Drugs* | 1 | -3.56694 | 2.12009 | -1.68 | 0.0936 | -7.74101 | 0.60713 |
| *Never used glucose monitoring* | 1 | 0.21020 | 2.04618 | 0.10 | 0.9183 | -3.81836 | 4.23876 |
| *Occasional glucose monitoring* | 1 | 2.58562 | 2.05668 | 1.26 | 0.2098 | -1.46361 | 6.63485 |
