# Supplementary material for: Supported Telemonitoring and Glycemic Control in People with Type 2 Diabetes: The Telescot Diabetes Pragmatic Multicenter Randomized Controlled Trial
Source: PLoS Med. 2016 Jul 26;13(7):e1002098. doi: 10.1371/journal.pmed.1002098 (PMC4961438; doi:10.1371/journal.pmed.1002098)
Supplement: S8 Table — (DOCX) [file pmed.1002098.s008.docx]

**S8 table: results of sub-group analysis for tertile of baseline HbA1c for the Telescot diabetes pragmatic randomized controlled trial**

| *Parameter Estimates – HbA1c (mmol/mol)* | | | | | | | |
| --- | --- | --- | --- | --- | --- | --- | --- |
| *Variable* | *DF* | *Parameter Estimate* | *Standard Error* | *t Value* | *Pr > \|t\|* | *95% Confidence Limits* | |
| *Intercept* | 1 | 63.23807 | 2.84913 | 22.20 | <.0001 | 57.62873 | 68.84741 |
| *Supported telemonitoring* | 1 | -2.88956 | 2.91407 | -0.99 | 0.3223 | -8.62675 | 2.84763 |
| *Subgroup 1* | 1 | 5.56998 | 2.92058 | 1.91 | 0.0576 | -0.18003 | 11.32000 |
| *Subgroup 2* | 1 | 13.14030 | 3.00701 | 4.37 | <.0001 | 7.22013 | 19.06046 |
| *Interaction 1* | 1 | -3.88660 | 4.05384 | -0.96 | 0.3385 | -11.86776 | 4.09455 |
| *Interaction 2* | 1 | -1.37368 | 4.13644 | -0.33 | 0.7401 | -9.51747 | 6.77010 |
| *Female sex* | 1 | 0.56341 | 1.77406 | 0.32 | 0.7510 | -2.92933 | 4.05615 |
| *Over 70 years old* | 1 | 2.91000 | 2.17125 | 1.34 | 0.1813 | -1.36473 | 7.18472 |
| *Centre: Lothian* | 1 | -1.42323 | 1.82110 | -0.78 | 0.4352 | -5.00860 | 2.16213 |
| *Centre: Glasgow* | 1 | 4.65957 | 4.02384 | 1.16 | 0.2479 | -3.26252 | 12.58165 |
| *Centre: Borders* | 1 | -9.90773 | 10.02433 | -0.99 | 0.3239 | -29.64352 | 9.82806 |
| *Two or more Diabetes Drugs* | 1 | -5.60184 | 1.94769 | -2.88 | 0.0043 | -9.43643 | -1.76725 |
| *Three or more Anti-hypertension Drugs* | 1 | -3.80335 | 2.14851 | -1.77 | 0.0778 | -8.03331 | 0.42661 |
| *Never used glucose monitoring* | 1 | -0.23065 | 2.06598 | -0.11 | 0.9112 | -4.29812 | 3.83682 |
| *Occasional glucose monitoring* | 1 | 2.77103 | 2.06326 | 1.34 | 0.1804 | -1.29109 | 6.83315 |
