# Supplementary material for: Supported Telemonitoring and Glycemic Control in People with Type 2 Diabetes: The Telescot Diabetes Pragmatic Multicenter Randomized Controlled Trial
Source: PLoS Med. 2016 Jul 26;13(7):e1002098. doi: 10.1371/journal.pmed.1002098 (PMC4961438; doi:10.1371/journal.pmed.1002098)
Supplement: S9 Table — (DOCX) [file pmed.1002098.s009.docx]

**S9 table: results of sub-group analysis for tertile of ambulatory blood pressure monitoring (ABPM) recording of systolic blood pressure for the Telescot diabetes pragmatic randomized controlled trial**

| *Parameter Estimates – Tertile ABPM Systolic blood pressure* | | | | | | | |
| --- | --- | --- | --- | --- | --- | --- | --- |
| *Variable* | *DF* | *Parameter Estimate* | *Standard Error* | *t Value* | *Pr > \|t\|* | *95% Confidence Limits* | |
| *Intercept* | 1 | 39.92313 | 5.40997 | 7.38 | <.0001 | 29.27186 | 50.57439 |
| *Supported telemonitoring* | 1 | -5.85260 | 2.74910 | -2.13 | 0.0342 | -11.26509 | -0.44012 |
| *Subgroup 1* | 1 | -0.34965 | 2.85233 | -0.12 | 0.9025 | -5.96538 | 5.26607 |
| *Subgroup 2* | 1 | 2.46268 | 2.95318 | 0.83 | 0.4051 | -3.35160 | 8.27697 |
| *Interaction 1* | 1 | 3.79588 | 3.96577 | 0.96 | 0.3393 | -4.01201 | 11.60377 |
| *Interaction 2* | 1 | -2.98092 | 4.01843 | -0.74 | 0.4588 | -10.89250 | 4.93066 |
| *Baseline HbA1c* | 1 | 0.40201 | 0.06361 | 6.32 | <.0001 | 0.27677 | 0.52726 |
| *Over 70 years old* | 1 | 3.02625 | 2.15403 | 1.40 | 0.1612 | -1.21465 | 7.26714 |
| *Female sex* | 1 | -0.15131 | 1.76332 | -0.09 | 0.9317 | -3.62298 | 3.32036 |
| *Centre: Lothian* | 1 | -1.53600 | 1.82027 | -0.84 | 0.3995 | -5.11978 | 2.04779 |
| *Centre: Glasgow* | 1 | 3.94786 | 3.99744 | 0.99 | 0.3242 | -3.92238 | 11.81810 |
| *Centre: Borders* | 1 | -11.57207 | 9.94317 | -1.16 | 0.2455 | -31.14841 | 8.00426 |
| *Two or more Diabetes Drugs* | 1 | -5.65287 | 1.92805 | -2.93 | 0.0037 | -9.44886 | -1.85688 |
| *Three or more Anti-hypertension Drugs* | 1 | -3.34697 | 2.13267 | -1.57 | 0.1177 | -7.54581 | 0.85187 |
| *Never used glucose monitoring* | 1 | 0.11934 | 2.03302 | 0.06 | 0.9532 | -3.88332 | 4.12200 |
| *Occasional glucose monitoring* | 1 | 2.35344 | 2.04642 | 1.15 | 0.2512 | -1.67559 | 6.38247 |
