# Supplementary material for: Supported Telemonitoring and Glycemic Control in People with Type 2 Diabetes: The Telescot Diabetes Pragmatic Multicenter Randomized Controlled Trial
Source: PLoS Med. 2016 Jul 26;13(7):e1002098. doi: 10.1371/journal.pmed.1002098 (PMC4961438; doi:10.1371/journal.pmed.1002098)
Supplement: S10 Table — (DOCX) [file pmed.1002098.s010.docx]

**S10 table: results of sub-group analysis for tertile of body mass index (BMI) for the Telescot diabetes pragmatic randomized controlled trial**

| *Parameter Estimates - BMI* | | | | | | | |
| --- | --- | --- | --- | --- | --- | --- | --- |
| *Variable* | *DF* | *Parameter Estimate* | *Standard Error* | *t Value* | *Pr > \|t\|* | *95% Confidence Limits* | |
| *Intercept* | 1 | 37.19048 | 5.49352 | 6.77 | <.0001 | 26.37471 | 48.00625 |
| *Supported telemonitoring* | 1 | -2.49841 | 2.83054 | -0.88 | 0.3782 | -8.07123 | 3.07441 |
| *Subgroup 1* | 1 | 3.24993 | 2.67320 | 1.22 | 0.2251 | -2.01312 | 8.51298 |
| *Subgroup 2* | 1 | 3.22495 | 3.14218 | 1.03 | 0.3057 | -2.96144 | 9.41134 |
| *Interaction 1* | 1 | -3.07082 | 4.02628 | -0.76 | 0.4463 | -10.99784 | 4.85620 |
| *Interaction 2* | 1 | -6.03005 | 4.15978 | -1.45 | 0.1483 | -14.21992 | 2.15981 |
| *Baseline HbA1c* | 1 | 0.41792 | 0.06378 | 6.55 | <.0001 | 0.29235 | 0.54349 |
| *Over 70 years old* | 1 | 3.10086 | 2.17248 | 1.43 | 0.1546 | -1.17636 | 7.37809 |
| *Female sex* | 1 | -0.16549 | 1.78460 | -0.09 | 0.9262 | -3.67905 | 3.34807 |
| *Centre: Lothian* | 1 | -1.48088 | 1.80421 | -0.82 | 0.4125 | -5.03304 | 2.07129 |
| *Centre: Glasgow* | 1 | 4.74142 | 3.98662 | 1.19 | 0.2354 | -3.10753 | 12.59037 |
| *Centre: Borders* | 1 | -10.39559 | 9.93068 | -1.05 | 0.2961 | -29.94733 | 9.15614 |
| *Two or more Diabetes Drugs* | 1 | -5.23822 | 1.92960 | -2.71 | 0.0071 | -9.03726 | -1.43918 |
| *Three or more Anti-hypertension Drugs* | 1 | -3.41408 | 2.12967 | -1.60 | 0.1101 | -7.60703 | 0.77887 |
| *Never used glucose monitoring* | 1 | -0.00517 | 2.04096 | -0.00 | 0.9980 | -4.02346 | 4.01313 |
| *Occasional glucose monitoring* | 1 | 2.59393 | 2.05233 | 1.26 | 0.2074 | -1.44675 | 6.63460 |
