# Supplementary material for: Supported Telemonitoring and Glycemic Control in People with Type 2 Diabetes: The Telescot Diabetes Pragmatic Multicenter Randomized Controlled Trial
Source: PLoS Med. 2016 Jul 26;13(7):e1002098. doi: 10.1371/journal.pmed.1002098 (PMC4961438; doi:10.1371/journal.pmed.1002098)
Supplement: S1 Text — (PDF) [file pmed.1002098.s011.pdf]

telescot

TELEmetric supported Self-monitoring of long-term COndiTions

The impact of a telemetric monitoring service in type 2 Diabetes. Randomised controlled trial with nested qualitative study

Protocol Version 3.7
29 August 2012

Tele-health programme

**Programme Lead**	**Professor Brian McKinstry**
Grantholders	Mrs Anne Reoch
	Dr Janet Hanley
	Mr Jim Forrest
	Prof John McKnight
	Professor William MacNee
	Professor Paul Padfield
	Dr Claudia Pagliari
	Dr Hilary Pinnock
	Professor Aziz Sheikh
	Dr Cathie Sudlow
	Prof Sarah Wild
Statistician	Dr Stephanie Lewis
Health economist	Dr Marjon van der Pol
Programme manager	Dr Lucy McCloughan

Diabetes trial

**Principal Investigator**	**Professor Brian McKinstry**
Diabetes group	Dr Janet Hanley
	Dr Stephanie Lewis
	Professor Brian McKinstry
	Prof Johnny McKnight
	Professor Paul Padfield
	Dr Claudia Pagliari
	Professor Aziz Sheikh
	Dr Marjon van der Pol
	Prof Sarah Wild
Collaborators	Mrs Anne Reoch
	Mr Jim Forrest
	Professor William MacNee
	Dr Cathie Sudlow
	Prof Anne-Louise Kinmonth
Trial Manager	Mary Paterson

**Funder**	Chief Scientist Office
	NHS Applied research programme grant

Contents

1.1 Self-monitoring in long-term conditions....3
1.2 The Telescot research programme....3
1.3 The trial of tele-monitoring in people with diabetes....4
2 Aims....6
3 Research Questions....6
3.1 Randomised controlled trial....6
3.2 Qualitative study....6
4 Outcome Measures....7
4.1 Primary outcome measure....7
4.2 Secondary outcome measures....7
5 Methods....7
5.1 Trial design....7
5.2 Setting....7
5.3 Participants....8
5.4 Sample structure and sample size....8
5.5 'Run in' Phase (Embedded Pilot)....9
5.6 Recruitment....9
5.7 Confirmation of eligibility and consent....10
5.8 Baseline measurements....10
5.9 Randomisation....10
5.10 Initial optimisation of care and self-management education....10
5.11 Protection against bias....10
5.12 Trial interventions....11
5.13 Follow up measures....12
5.14 Safety....13
5.15 Data collection and statistical analysis....13
5.16 Qualitative study....14
6 Exit Strategy....15
7 Publication Strategy....15
8 Risk Management Strategy....15
8.1 Data security within the telemetry system....15
8.2 Contamination between the intervention and control groups....16
8.3 Home monitoring revealing problems which require intervention....16
8.4 Excess workloads for practices....16
8.5 Equipment Failures....16
10 Project Management and Quality Assurance....17
11 Timescale....17
12 Reporting....17
13 Finance....17
14 References....17
15 Appendices....22
15.1 Model of telemetry supported self care....22
15.2 Rapid Treatment Protocol....23
15.3 Type 2 Diabetes Blood-glucose-lowering therapy....24
15.4 Theoretical framework for telehealth studies....28
15.5. Timescales....31

1. Introduction

1.1 Self-monitoring in long-term conditions

Long-term conditions are a major healthcare challenge.[1] As the population ages more people are living with long-term conditions rendering the current, clinician-centred models of management unsustainable in the longer-tem.[2] NHS policy increasingly encourages people to self-manage their condition with additional professional support for those at greatest risk.[3] Systematic reviews indicate that engaging patients in self-monitoring and management can improve clinical outcomes in asthma[4] but the evidence that self-monitoring alone is beneficial in hypertension[5] and other chronic conditions including diabetes[6] is equivocal. This may be because adherence to both lifestyle advice and prescribed medication for these groups is poor generally [7] but also because feedback from clinicians is often infrequent, potentially adding to patient anxiety in the presence of abnormal self-monitored readings.[8]

Monitoring is, in itself, an intervention which alters behaviour. The theoretical model developed by Glasziou *et al.* describes the complementary and evolving roles of periodic professional support and on-going patient self-monitoring.[9] This approach resonates with two key health service policies: the drive for technological solutions to healthcare problems [10]; and the importance of expert patients and self-management of long-term conditions[11,12] While some evidence can be gleaned from international research into telemetric solutions for chronic disease management and the encouraging results from UK and international pilot studies[13-17], there is little research into the cost-effectiveness of telehealthcare interventions[18] and there remains a pressing need to evaluate rigorously such interventions in a UK NHS setting.

Effective interventions need to reflect patients' needs, enable professionals to adapt their working practices and address organisational infrastructure. Telemetric systems enabling patient self-monitoring and relay of information to clinicians can potentially improve integration between self-management and professional support, as well as facilitate intensive monitoring and aggressive intervention when needed.

The service model to be tested is shown in Appendix 15.1. The systems can work on a range of technology platforms and with multiple measuring devices, facilitating seamless care for patients with several co-morbidities. The Telescot research programme focuses on four disease areas which account for much long-term morbidity in the population, a high proportion of consultations in primary care and are major causes of hospitalisation, namely: chronic obstructive pulmonary disease (COPD); hypertension; blood pressure reduction after stroke/transient ischaemic attack; diabetes and obesity.

1.2 The Telescot research programme

Our programme of work sits within the MRC framework for the development and evaluation of complex interventions.[19] Building on existing literature[13;20;21], and in an iterative process using insights from completed and on-going exploratory and pilot work[22], we have designed Phase III randomised controlled trials using a suggested framework for complex interventions.[23] These will evaluate how telemetry aided, supervised self-monitoring affects the management of long-term conditions in four different contexts (largely asymptomatic conditions using the example of hypertension; symptomatic, potentially unstable and progressive conditions using the example of COPD; an older, more disabled group with challenging management targets using the example of hypertension among stroke survivors; co-morbid conditions using the example of diabetes, hypertension and weight management). Each will incorporate qualitative and quantitative process evaluations, which are increasingly recognised as being critical to the subsequent implementation. [24;25]

The following will be performed for each condition:

- A randomised controlled trial to evaluate the clinical effectiveness of telemetrically supported self-monitoring
- A qualitative study to improve understanding of the impact of telehealthcare interventions on patients, carers and healthcare professionals, and to explore the facilitative factors and barriers to implementation in patients and practices.

- Health economic analysis from the perspective of both the NHS and the patient, to provide the evidence on cost-effectiveness and to inform the role of telehealthcare in the NHS

1.3 The trial of tele-monitoring in people with diabetes

There are over 200,000 people in Scotland with diabetes and this figure is expected to rise to 350,000 by 2030. [26] Approximately 14% of people with type 2 diabetes on the Lothian diabetes register in 2008 had both poor control of glycaemia (HbA1c$\geq$7.5%) and systolic blood pressure above the National Institute of Health and Clinical Excellence (NICE) target (140 mmHg). Annual health care costs of a patient with Type 2 diabetes are over six times higher than the costs of a person without, largely due to the development of diabetes-related complications. Control of blood glucose and blood pressure (as well as management of dyslipidaemia) among people with diabetes reduce complications and mortality.[27-29]

Poor glycaemic control and raised blood pressure are largely asymptomatic so people with diabetes who are not self-monitoring their glucose have little feedback on the impact of lifestyle changes or their medication between surgery visits. These factors probably contribute to the fact that adherence to lifestyle advice is generally poor.[7] Effective self-management of diabetes also involves a complex combination of behavioural changes, integrating regular self-testing with dietary and exercise regulation, and this can be challenging and stressful for patients. [30]

The current medical model for managing blood pressure in people with diabetes and in primary care is costly, does not necessarily engage individuals in a self-care approach, and may not lead to treatment being well targeted both because ‘one off’ measurements in the surgery are poor predictors of average blood pressure and as a consequence of clinicians’ failure to change or increase treatment when blood pressure targets are not met (known as therapeutic inertia).[31;32] Current care is costly because hypertension is common: almost 13% of people registered with a GP in Scotland are hypertensive according to 2007/8 Quality Outcomes Framework data.[33] Diabetes is also common and diabetes and hypertension commonly co-exist. Prevalence of diabetes and hypertension can be expected to increase in association with ageing of the population and increasing numbers of people who are overweight or obese. Experts now advocate more intensive risk identification and management of hypertension than has been used in the past[34] including reducing the threshold at which intervention should start, particularly among people with diabetes. This cannot easily be accommodated within the current model of service due to increases in both the numbers of consultations per patient required to achieve ambitious management targets and the numbers of people defined as hypertensive. Other models will need to be considered and one advocated in current NHS policy is self-care, where the individual takes greater responsibility for his/her health.[35]

A growing private market for home blood pressure monitors[36] suggests that many patients do want to monitor their own blood pressure; there is furthermore (as discussed below) considerable interest in glucose self-monitoring among people with diabetes. Lack of NHS support for modifying treatment based on the results of self-monitoring may therefore be a missed opportunity to engage people in improving self-care. Telemetrically supported monitoring has been shown in small studies in other health care systems to improve glycaemic control among people with diabetes.[13;14;37] Telehealth also offers opportunities to help patients cope with the challenges of self-care through the provision of remote advice and support, as demonstrated in a recent trial of a tailored text-messaging service.[35] It is important to determine to what extent control of diabetes and blood pressure may be achieved in NHS primary care using this technology.

A well recognised difficulty for those attempting to identify and manage patients with high blood pressure is its inherent variability. Single measurements taken in the surgery are poorly correlated with average blood pressure measured by either ambulatory recording or home monitoring.[30] This is important because there is evidence that average ambulatory blood pressure is a better predictor of cardiovascular morbidity and mortality than surgery readings

and growing evidence that average blood pressure based on home monitoring is also superior to office readings.[31] Guidance to professionals recognises the possibility of false positives when checking blood pressure and recommends that a diagnosis of hypertension is only made if the measurement is over 140/90 mmHg on three occasions. [38] The possibility of false negatives is not routinely taken into account and a 'normal' blood pressure is unlikely to be rechecked either in a screening situation or as part of ongoing management. Indeed the Quality and Outcomes Framework, which determines general practitioner (GP) payments now provides a financial disincentive for doing so because a subsequent high measurement in the record may lead to reduced practice payments. In public health terms, the need to check blood pressure on several occasions to establish that it is consistently high is burdensome to patients and professionals and is likely to slow treatment changes needed. Moreover, some people (an average of 17% in a recent meta-analysis) will have been falsely reassured that their blood pressure is well controlled when they have "masked" hypertension. [39]

Self-monitoring has been suggested as a possible means of providing good quality data which will correlate well with outcomes. However, the evidence that self-monitoring alone is beneficial in hypertension and other chronic conditions is equivocal. The most recent Cochrane review suggests that home monitoring without telemetry can lead to falls in diastolic blood pressure of 2-3mm Hg but only a non-statistically significant trend for reduction in systolic blood pressure; and also that intensive monitoring and treatment can lead to falls in systolic blood pressure of more than 5mm Hg. [5] However, the meta-analysis which led to the latter finding was dominated by a very large trial carried out in the 1970s in the United States (US)[40] and the applicability to a UK context and modern medication regimens cannot be assumed.

A systematic review and meta-analysis of six randomised controlled trials (RCTs) concluded that self-monitoring of blood glucose (SMBG) in patients with type 2 diabetes not using insulin was associated with a small, but statistically significant decrease in HbA1c levels (of 0.39% (95% CI 0.21 to 0.56), which was considered clinically relevant because findings from the UK Prospective Diabetes Study (UKPDS) suggest that this decrease in HbA1c would be expected to reduce microvascular complications by approximately 14%.[41] However the authors commented that their findings should be interpreted with caution as a consequence of the poor quality of the trials and the heterogeneous study populations and interventions. A recent UK trial of self-monitoring of glucose among 453 people with non-insulin treated diabetes reported a difference in unadjusted mean change in HbA1c level from baseline to 12 months between the control and more intensive self-monitoring groups of -0.17 (95% CI -0.37 to 0.03%).[6] It is possible that the apparent lack of effect of self-monitoring may reflect failure to act on the results perhaps because health professionals do not receive the results and/or do not follow treatment guidelines. In the US, observational studies of telemetry in diabetes demonstrated reductions in clinic visits and hospitalisation.[37] RCTs of telemedicine and web-based management showed reductions in HbA1c [37] and systolic BP[14] in the intervention compared to comparison groups, but a study in children with type 1 diabetes found no effect of tele-communication technology on glycaemic control.[15] A small randomised controlled trial performed in the US of tele-monitoring of weight and biweekly telephone counselling over six months found that immediate intervention was effective at producing weight loss compared to both no treatment and to delayed intervention.[42] Weight loss is known to improve both glycaemic and blood pressure levels, but is challenging to achieve, particularly as improved glycaemic control is often associated with weight gain.

Explanations for why self-monitoring has not been as successful as had been hoped in long-term condition management includes patients perceptions that professionals pay little heed to their results and because long periods can pass before data are reviewed. There is also concern that home blood pressure monitoring or self-monitoring of blood glucose (SMBG) may make some people anxious.[8;43] Telemetry and tele-healthcare offer a potentially convenient means of supporting patients with diabetes, offering opportunities to harness the benefits of reminder systems (thus encouraging self-testing), biofeedback (enabling patients to 'see' the impacts of lifestyle and medication compliance) and remote support from professionals or peers (e.g. medication changes, alerts or motivational messages). While these features have potential to improve patients' blood pressure and associated health

outcomes, evidence for the clinical benefits of telemetric monitoring for diabetes and blood pressure is still limited and further research is urgently needed to fill this knowledge gap.[44]

The introduction of a nurse led, telemetric, home blood pressure and glycaemic control monitoring service could potentially address a number of these issues. It is anticipated that the intervention will work at two levels. The first is at the patient level. Patients will have control of the measurement of their blood pressure and blood glucose. They will be able to get timely feedback on the impact of changes to lifestyle and medication, reminders, alerts, on-line information and they will also be able to contact their practice nurse (who will be able to see their blood pressure and glucose record on the internet) via telephone or email. Patients will still attend annual diabetes reviews but will not have to visit the practice for routine blood pressure checks. It is anticipated that this will provide increased motivation and facilitation for individuals to improve self-care. The intervention may also work at a professional level. Professionals will have remote access at any time to a reliable estimate of average blood pressure and SMBG levels. Therapeutic inertia arises from the fact that professionals generally tend to wait for repeated abnormal readings often over several months before suggesting changes to treatment. The availability of the home monitoring results may give professionals more confidence in their assessment of the effectiveness of the patient's self-care and medication and lead to effective regimens being established more quickly.

Further research is required, particularly in an NHS setting, to establish the role of supported blood glucose, blood pressure and weight monitoring among people with poorly controlled type 2 diabetes.

2 Aims

To investigate the clinical effectiveness, and the social and service impact of introducing telemetry-aided, supervised, self-monitoring for type 2 diabetes.

3 Research Questions

3.1 Randomised controlled trial

In people with poorly controlled type 2 diabetes (i.e. HbA1c > 7.5% or >58 mmol/mol) does supported telemetric monitoring compared to usual care:

1. Reduce HbA1c?
2. Reduce average blood pressure?
3. Reduce weight (in overweight people)?
4. Improve adherence to lifestyle advice (smoking, alcohol and salt intake, physical activity)?
5. Improve disease-specific quality of life?
6. Reduce anxiety and depression?
7. Improve patient knowledge and self-efficacy?
8. Engage patients in self-care and improve compliance?
9. Represent a cost-effective use of NHS resources?

3.2 Qualitative study

1. What are the experiences and opinions of people with type 2 diabetes of this service (including impact on behaviour, mood, positive and negative experiences and change in relationship with their healthcare provider)?
2. What are healthcare providers' experiences and opinions of this service?

4 Outcome Measures

4.1 Primary outcome measure

- Difference in mean HbA1c between intervention and control groups measured at nine months.

4.2 Secondary outcome measures

- Difference in mean daytime systolic BP measured by ABPM between intervention and control groups measured at nine months.
- Difference in mean daytime diastolic BP measured by ABPM between intervention and control groups measured at nine months.
- Difference in mean weight between intervention and control groups measured at nine months.
- Incremental cost-effectiveness measured as Cost per Quality Adjusted Life Year (QALY)

The following variables will also be measured at baseline and follow-up:

- Surgery measured blood pressure
- serum total cholesterol (from random, probably non-fasting sample)
- exhaled carbon monoxide (to verify self-reported smoking status)
- urinary sodium/creatinine ratio (to reflect salt intake)
- renal function (eGFR)
- UKPDS risk score (using data on age, sex, blood pressure, smoking, cholesterol, and HbA1c)[45]
- self-reported alcohol intake and smoking
- anxiety and depression (HADS)[46]
- quality of life (EQ-5D)[47],
- self-efficacy (short version of chronic disease self-efficacy scale)[48]
- medication adherence[49]
- self-reported physical activity (International Physical Activity Questionnaire)[50]
- self-reported exercise tolerance[51]
- knowledge of managing diabetes[52]
- questionnaire to assess the participant's ethnic group, taken from the 2011 Census [53].
- number of attendances at practice nurse, GP, accident and emergency, out of hours care, and hospital admissions
- prescriptions for anti-hypertensives, diabetes medications and test strips, lipid-lowering drugs and obesity drugs taken from GP practice records
- number of telephone / email contacts with practice nurses and GPs.

5 Methods

5.1 Trial design

A 9 months researcher-blinded randomised controlled trial.

5.2 Setting

A multicentre study which will run in Primary Care in selected practices across the UK including practices in NHS Lothian, NHS Borders, NHS Highland and Kent.

5.3 Participants

5.3.1 Inclusion criteria

Patients registered with general practices in the selected areas who:

- are on their practice diabetes registers;
- have type 2 diabetes which is managed mainly in general practice;
- are aged 18 years or over;
- have a last recorded HbA1c measurement >7.5% (>58 mmol/mol);
- have given informed consent;
- have a mobile telephone signal available from home.

5.3.2 Exclusion criteria

Patients registered with general practices in the selected areas who:

- Have an average blood pressure >210/135 taken by the research nurse at the consent visit;
- have HbA1c≤7.5% (<59 ≤58 mmol/mol);
- have hypertension or renal disease being managed in secondary care;
- have had treatment for a cardiac event, or other life-threatening illness within the past 6 months or have had major surgery within the last 3 months;
- are unable to consent;
- are unable to use self-monitoring equipment;
- have atrial fibrillation unless successfully treated or cardioverted;
- are pregnant.

5.4 Sample structure and sample size

There were 4440 people with type 2 diabetes, an HbA1c of >7.5% and systolic blood pressure of ≥135mmHg on the Lothian diabetes register in 2008. This group reflects approximately 16% of people on the register with type 2 diabetes. Their mean (SD) HbA1c was 8.8% (1.4%) and their mean (SD) systolic blood pressure was 149 mmHg (13 mmHg).

Using these values, we calculate that a randomised controlled trial with 125 people completing each arm with no change in values in the control group at the end of the trial would have 80% power at 5% significance to detect a 0.5% absolute fall in HbA1c and a 5 mmHg fall in systolic blood pressure in the intervention group.

We will recruit approximately 25-30 socially diverse practices to the trial using both the English and Scottish Primary Care Research Networks. We will make use of the network of practices in NHS Lothian we have established during our HITs blood pressure trial, who are familiar with the technology and have already expressed a willingness to take part. We will also use the networks and contacts provided by our grantholders to extend recruitment beyond NHS Lothian, NHS Borders and NHS Highland. At present we are planning to recruit from four large practices in Kent. The practices we will recruit will already have nurse-led diabetes clinics run either by nurse prescribers or by non-prescribers and will use protocols (based on NICE Guidelines) for arranging changes to anti-hypertensive medication and diabetes treatment respectively. Training for the practices will be provided by the researchers.

Three hundred and twenty (320) patients will be recruited from participating practices. Practice diabetes registers will be searched for potentially eligible patients and the list generated will be checked by GPs for exclusion criteria not readily available from the electronic record. The GP will send a letter to potential participants outlining the study and asking if they wish to express an interest in participating in the study. Reminders will be sent to patients after 10 days. Patients expressing an interest will be invited to attend a baseline assessment at their practice.

In an earlier trial of telemetric monitoring of blood pressure[54] 60% of patients on a practice hypertension register agreed to take part and 82% were retained within the trial; we anticipate similar recruitment and retention rates in this study. We therefore plan to recruit a total of approximately 320 patients to ensure two final group sizes of 125 people which will provide sufficient power (assuming that up to 20% of people may drop out of the trial).

According to data held in NHS Lothian, an NHS Lothian practice with an average list size of 6000 patients will have approximately 210 patients with type 2 diabetes (given a local diabetes type 2 diabetes prevalence of 3.5%). Based on the proportion of people with non-ambulatory systolic blood pressure ≥135mmHg and HbA1c>7.5% (≥ 59 mmol/mol) on the Lothian diabetes register in 2008 (16%) and the assumption that a third of these people will already be on insulin, we expect approximately 23 people per practice will meet the inclusion criteria. (Note that Type 2 diabetics on insulin are not generally managed in primary care in NHS Lothian and so will not meet our inclusion criteria.) Assuming that 60% of patients are willing and able to take part we would expect to recruit approximately 14 people per practice.

We have also run preliminary searches on a large practice (32000 patients) in Kent. This has identified approximately 140 individuals who meet the inclusion criteria. Assuming that 60% of patients are willing and able to take part we would expect to recruit approximately 80 patients from this practice. (Note that Type 2 diabetics on insulin are managed in primary care in this practice and are therefore eligible for inclusion.)

Our estimate of the requirement for 25-30 practices required overall is based on our experience with HITS, and reflects a conservative approach to estimating the numbers who are likely to take part. The number of practices required may be smaller than this and will depend on the actual list sizes of selected practices as well as the numbers of eligible patients actually identified in the specific searches.

The practices recruited will all already have nurse-led diabetes clinics with support available from a general practitioner with an interest in diabetes. Training for the practices including the use of protocols based on the Lothian Hypertension Guideline (see Appendix 15.2) and/or NICE guidelines for managing Type 2 diabetes agreed for the study (see Appendix 15.3) will be provided by the researchers.

The sample size has been estimated to allow for up to 20% drop out. However, every effort will be made to maximise retention. Patients may stop using the intervention whenever they wish but will be encouraged to have their follow-up data collected at the end of the trial regardless of their adherence to the intervention to enable their data to be included in the planned intention-to-treat analysis.

5.5 'Run in' Phase (Embedded Pilot)

A 'run in phase' (embedded pilot) will take place in Blackburn Health Centre (the Chief Investigator's practice) to test the feasibility of all aspects of the trial process including communication, training, recruitment, consent, randomisation, and data collection. Given that there are concerns about whether it will be possible for participants to take weighing scales home from the practice and to use the scales in an appropriate fashion, particular attention will be given during this run in phase to the feasibility of collecting information about participants' weight.

5.6 Recruitment

Potentially eligible patients will be sent the study materials by their general practice with a covering letter and asked to contact the researcher if they are interested in participating. A second invitation will be sent out to non-responders after 10 days. We intend to recruit around 10 people per week over an 8 month period.

5.7 Confirmation of eligibility and consent

Once a patient has indicated interest in participating they will be invited to a clinic in their GP's surgery run by a research nurse (Wellcome Trust Clinical Research Facility (CRF) or equivalent). At the initial appointment the nurse will go through the study information with the patient, take written informed consent (which will cover consent to have their eligibility checked as well as their consent to take part in the trial), check potential eligibility, ask the patient to complete a short questionnaire on their demographic characteristics, past and current self-monitoring of blood pressure and blood glucose, collect a blood sample for HbA1c and cholesterol measurement and initiate ambulatory blood pressure monitoring using the Spacelab Ambulatory Blood Pressure Monitor.[55] The nurse will also explain the process for recruitment and potential randomisation if patients are eligible.

All patients will be offered the loan of an ABPM with which they can monitor their blood pressure for 14 hours. This will benefit the patient in that they will have an accurate estimation of their blood pressure whether or not they subsequently decide to ahead with the trial or are ineligible, however, if they wish they can delay this until after the result of their HBA1C is available.

Once the HbA1c result is available, any ineligible patients will be telephoned to let them know that they do not need to return for a second appointment to gather baseline measurements.

Patients' ABPM results will be released to the practice.

5.8 Baseline measurements

On returning for their second appointment, participants will have the following baseline measurements taken: smoking, height and weight, and exhaled carbon monoxide. In addition they will be asked to complete questionnaires as listed in section 4 above, as well as a questionnaire to assess the participant's ethnic group, taken from the 2011 Census [53].

5.9 Randomisation

All eligible participants will be randomised using the randomisation service run by Edinburgh Clinical Trials Unit. We will use minimisation with a random element based on age (below 70 or 70 and above), sex, location (i.e. Lothian, Borders, Highland or Kent) use of two or more diabetes drugs, use of three or more hypertension drugs and glucose self-monitoring history (never, occasionally, regularly) in order to make sure that these factors are distributed equally between intervention and usual care groups. The allocated treatment code will be generated by the Edinburgh Clinical Trials Unit. The participant will then be informed about the arm of the trial to which they have been assigned and given appropriate information and demonstration of equipment. All allocated patients will be included in the intention-to-treat analysis.

5.10 Initial optimisation of care and self-management education

All patients will be seen by a healthcare professional trained in the management of type 2 diabetes who will assess current control, optimise management in line with Lothian or NICE guidelines (see Appendix 15.1 and 15.2) and deliver a one-to-one standardised education session.

5.11 Protection against bias

Baseline data collection will take place prior to randomisation and allocation which will be carried out remotely at the Clinical Trials Unit to ensure adequate concealment. It is not possible to blind clinicians or patients to allocation thus potentially introducing bias in subsequent care. However, baseline data collection will be undertaken by the research

nurses before randomisation takes place; and final data collection will be undertaken as far as possible by a different research nurse to the one who undertook the initial assessment who will therefore be blinded to the allocation. Patients will be requested not to reveal their allocation, although we recognise that inadvertent references by the patients or in their primary care record may reveal their allocation. The use of objective outcomes (HbA1c, ambulatory blood pressure, weight validated questionnaires) will also reduce the possibility of bias. Patients will be asked not to reveal which group they are in and will be asked to return their equipment (if they are in the intervention arm) to the practice reception prior to the follow up appointment with the research nurse. The research nurses will be asked to report if they have been unblinded prior to the final assessment.

5.12 Trial interventions

5.12.1 Intervention group: Telemedicine monitoring

Patients in the intervention group will be given blood pressure and blood glucose monitors and weighing scales which use Bluetooth to transmit readings via a (supplied) modem to a remote server. The user may securely access their record on the server at any time (either at home if they have internet access, or in a library or other public internet access point). Their GP and practice nurse will also be able to access this record via the Internet. Users will also receive regular (monthly) feedback based on their blood pressure and blood glucose over the past 10 readings which will be sent by post or email according to the patient's wishes. The system can be set to provide reminders to check blood glucose, blood pressure and weight to both the user (via the user's mobile phone) and the GP practice (via email) when measurements remains high.

Users of this service will be trained in its use and the demonstration will be backed up by written information. The written information pack will also contain leaflets about initiating or maintaining lifestyle changes and how long it is likely to be before any lifestyle or medication change will have an impact on their rolling average blood pressure or blood glucose. The suggested lifestyle changes will include weight control, reducing salt in the diet and increasing physical activity.

The user will also be given contact details for the practice nurse and encouraged to make contact by email or by telephoning at specified times if their blood pressure or blood glucose remains high, to discuss their treatment. In the event of a very high blood pressure (>220 mmHg) or very high blood glucose levels (>15mmol/l) patients will be asked to repeat the measurements and if they remain high patients will be advised to contact their practice urgently for advice. Patients recording very low blood glucose (<4mmol/l) will be advised to eat or take a glucose drink and, if hypoglycaemia persists, to contact the practice. Practice nurses will be encouraged to review the alerts they are sent about patients whose blood pressure or blood glucose remains high or persistently low or who have not checked their blood pressure or blood glucose within the agreed timeframe on a weekly basis. Practice nurses may choose to contact the patient to offer further treatment or support and will be asked to record such contacts in the normal practice electronic record. The nurses will also formally review each record at an agreed time to transfer the current average blood pressure and blood glucose into the primary care record.

Patients will be encouraged to check their blood pressure 10-20 times over the first seven days to establish a reliable average and then about 4 times a month if their average blood pressure is at or below the target of 130mmHg. If blood pressure is above the target and any changes to lifestyle or medication are made, the users will be asked to undertake another more intensive period of monitoring (10-20 readings over seven days) after an appropriate time (usually 3-4 weeks depending on medication change) in order to establish the impact on the rolling average blood pressure. The guidance will not be restrictive as this tool is being provided for patients to use in order to understand their blood pressure better and to better manage their own health.

Patients in the intervention group will be asked to provide at least twice weekly measurements of morning and evening blood glucose and weekly measurements of morning weight. These measurements will be automatically forwarded to the study website and checked at least weekly by the practice nurse. Patients with high readings will be highlighted by the software for easy identification. The nurses will contact the patients by telephone, text or email to adjust therapy and reinforce lifestyle advice on the need for more or less intensive monitoring. Anti-hypertensive drug treatment may be altered every 4-6 weeks until mean BP based on the last 10 readings is at or below the target of 130mmHg. Treatment for diabetes will be altered according to the protocol every 4 weeks with the aim of maintaining consecutive fasting blood levels between 4 and 6mmol/l. Patients who require the addition of insulin to their treatment during the course of the trial will be jointly managed by the practice nurse and general practitioner along with specialist diabetes nurses at the local hospital (if appropriate), all of whom will have access to the telemetric data for patients in the intervention group. Algorithms for BP and glucose management are given in Appendix 15.2 and Appendix 15.3 respectively.

Within GP practices, no additional infrastructure is required, but practice nurses will need to set aside some time to answer telephone/ text/ email queries from patients, and will require access to the records on the remote server. This time should be offset by a reduction in face-to-face consultations for blood pressure checks. This way of working represents a major change for practice nurses in that it is the patient who knows what their blood pressure is, decides when to recheck it and decides when to contact a professional.

5.12.2 *Control group: paper-based monitoring*

Patients in the control group will receive normal care attending the practice nurse or GP for face-to-face appointments in the usual way. They will be asked about their use of self-monitoring of blood pressure and blood glucose at baseline and follow-up. Patients with uncontrolled blood pressure in our recent audit saw the nurse or doctor 3-4 times on average in 6 months and we would anticipate a similar number of attendances for patients with both uncontrolled glycaemia and blood pressure. Patients requiring insulin during the course of the trial will be managed jointly as above with specialist diabetes nurse support.

5.12.3 *Clinical care*

Throughout the trial, patients will be reviewed according to clinical need by their normal clinical advisors. Clinical care in both groups for patients in NHS Lothian will be in accordance with NHS Lothian protocols which are based on the recommendations of national and international guidelines, and in other areas according to NICE guidance. Therapeutic algorithms for both controls and intervention will follow guidelines (see APPENDIX 15.3), based upon the NICE guidelines [56] using a standard stepped treatment programme for blood glucose.

5.11.4 *Duration of intervention*

Nine months.

5.13 Follow up measures

Participants will be contacted 4 weeks before their follow up appointment is due with a reminder that an appointment letter will be sent to them in the next fortnight. At the follow up appointment, participants will be seen by a researcher / research nurses blinded to their allocation at their GP practice 9 months after randomisation. There they will complete questionnaires, have blood taken (for HbA1c and cholesterol) and be fitted for ABPM. They will return the ABPM monitor to the practice reception the following day. The results from the ABPM will be sent to the patient following download of the data. Use of healthcare resources

(number and duration of hospital admissions, including whether primary diagnosis was diabetes-related, practice and out-of-hours consultations, routine reviews for BP or diabetes, prescriptions for anti-hypertensive and diabetes drugs and test-strips) and adverse events will be extracted from the participant's electronic record by the researcher or research nurse at the follow up appointment. Electronic records will be retrieved and analysed for compliance with monitoring. The baseline measures (excluding height) will be repeated at the end of the 9 month follow-up period.

5.14 Safety

Adverse events which are related to diabetes, hypertension, the trial devices or anything else that causes concern will be recorded on Study Specific Adverse Event Forms. The assessment and notification of adverse events will be made via the following routes:

- Research nurses will ask all patients about adverse events at the follow up appointment;
- Practice nurses can report adverse events following communication with any patients whose blood pressure or blood glucose results give rise for concern;
- Any other potential adverse events which are alerted to the Trial team will be referred to the Chief Investigator to determine their status.

All adverse events will be rated by the Chief Investigator for seriousness where serious adverse events are defined as any untoward medical event that:

- results in death
- is life-threatening
- requires hospitalisation or prolongation of existing hospitalisation
- results in persistent or significant disability or incapacity
- consists of a congenital anomaly or birth defect

Any events rated as serious will then be rated for relatedness (unrelated to the trial intervention, possibly related to the trial intervention, probably related to the trial intervention or definitely related to the trial intervention) and expectedness (expected or unexpected). Events will not be followed up beyond the final visit. The outcome will be recorded on the database as recovered, ongoing or unknown.

Any SAEs which are rated as related (possibly, probably or definitely related) to the trial intervention and unexpected will be reported to the Ethics Committee using the NRES Safety Report Form (non CTIMP), within 24 hours of the trial team being made aware of the event.

A summary of all adverse events and serious adverse events will be presented to the Independent Trial Steering Committee every six months

5.15 Data collection and statistical analysis

Records will be kept of the numbers of practices and patients that were approached to participate in the study and the numbers and proportions of practices and patients that consented or declined to take part. Data from participating individuals will be entered from questionnaires and paper records by the researcher to a database with a double entry function. A random 10% sample checked for accuracy by other trial staff. If we detect systematic errors we will re-enter all the data. Every attempt will be made to collect complete data on all of participants.

Data analysis will be carried out by the Edinburgh Clinical Trials Unit, supervised by Dr Steff Lewis. Analysis will be on a modified intention to treat basis by an individual who is blinded to group allocation. In the primary analysis we will remove patients with missing outcome data, but will perform sensitivity analyses to investigate the effect of this. Binary outcomes will be presented both as relative and absolute risk reductions. For HbA1c, and blood pressure and other normally distributed measurements that are taken at baseline and follow-up, adjustment

for baseline measures will be performed using analysis of covariance. For the primary outcome, subgroup analyses will be performed based on sex, tertiles of age and socio-economic status (based on the Scottish Index of Multiple Deprivation derived from postcode) and tertiles of baseline HbA1c, home self-monitored systolic blood pressure and body mass index. These factors may all reasonably be hypothesised to affect the uptake of the intervention. Subgroup analyses will be performed by adding any interaction between these factors and treatment into the analysis of covariance model and observing whether the fit of the model is statistically significantly improved. No interim analysis is planned.

The health economic analyses will assess the cost-effectiveness of the tele-supported self-monitoring compared to usual care. A cost-utility analysis (incremental cost per QALY) will be performed. The perspective will be the NHS. The benefits will include health outcomes measured in terms of QALYs which will be derived from the responses to the EQ-5D.[50] Health service (GP/nurse consultations, telephone consultations, home visits, accident and emergency attendances, out-patient consultations, hospitalisations) and drug use over the trial period will be extracted from practice records to check the validity of self-reporting. The costs of the tele-monitoring equipment and the set-up and support costs will be estimated using expert judgment. Resource use estimates will be combined with unit costs obtained from standard sources such as the Personal Social Services Research Unit.[57] The results of the economic evaluation will be presented as an incremental cost-effectiveness ratio (cost-utility analysis). The evaluation will include both deterministic and probabilistic sensitivity analysis. Modelling of longer term costs and benefits will be explored using estimates from published studies including UKPDS.[58] Advice on the health economics analysis will be provided by Dr Marjon van der Pol from the Health Economics Research Unit at the University of Aberdeen.

5.16 Qualitative study

5.16.1 Patients

A sub-sample of participants in the randomised controlled trial will be recruited to the nested qualitative study. Up to 20 participants from the intervention group will be recruited for semi-structured interviews plus a further 10-12 for two focus groups. The purpose of the focus groups will be to allow the group to explore shared ideas and experiences relating to type 2 diabetes and their experiences in managing it. The interviews with individuals will cover similar subjects but will allow private discussion of issues such as not using medication in the prescribed way (which people may not be willing to discuss in a public situation). A maximum variation sample in relation to age, social class, ethnicity (if ethnicity varies sufficiently between participants) and severity of type 2 diabetes, plus level of use of the system will be sought.

5.16.2 Healthcare Professionals

Up to 20 professionals participating in the trial will be interviewed (face-to-face or by telephone according to the preference of the clinician). This will include the specialist diabetes nurses providing the support services, long-term condition nurses, and representatives of primary and secondary care services.

5.16.3 Topic guides

The initial guide for semi-structured interviews with patients will be based on the themes identified from the literature and the on-going studies, but the guide will be reviewed and refined iteratively as data are gathered and analysed and new themes arise. Participants will therefore be encouraged to give their views on the usefulness of the systems in general and then tell their own story about managing their own long-term conditions and the impact of the systems.

Interviews with professionals will seek to investigate perceptions of the benefits (or otherwise) of the intervention, experiences of implementing and maintaining it and the barriers and facilitators they have experienced.

5.16.4 Analysis

The interviews and focus groups will be fully transcribed. The Framework method will be used to classify and organise data according to key themes, concepts and emergent categories.[59] Data will be analysed from the theoretical perspective of the diffusion of innovations literature and the behaviour change literature, underpinned by social learning theory, which emphasise the importance of people's perceptions in understanding their behaviour in relation to an innovation.[60-64]

Initial coding will be carried out by the qualitative researcher with reference to the transcripts and voice recordings and the analysis recorded using NVivo 7. Constant comparison (checking experiences against those of others in the sample) will ensure that the thematic analysis represents all perspectives and negative cases will be sought. The analysis will then be reviewed by the wider research team to aid interpretation. Validity checking of the analysis will include recoding of some interviews by an independent researcher and coding review of some of the data by a patient reference group.

6 Exit Strategy

It will be clear in the study information that the patient will be expected to return the equipment after 9 months and that their practice will not be able to support this type of service outside the trial. However, it will be explained to patients that while this is an experimental procedure, if it is found to be cost effective in the future it is likely that the NHS will adopt it.

7 Publication Strategy

The findings of this trial will be published in peer-reviewed journals, presented as abstracts at national and international conferences and disseminated via the co-applicants contacts with professional and policy bodies.

8 Risk Management Strategy

The main risks associated with this study are data security within the telemetry system, contamination between the intervention and control groups, home monitoring resulting in the identification of problems (e.g. extremes of blood pressure or blood glucose measurement or onset of atrial fibrillation) which need intervention and create excess workloads for practices, and equipment failures.

8.1 Data security within the telemetry system

The clinical data collection system will held in a secure server at the IEM GMbH in Germany (the provider of the Telehealth Service). Access to the data will be by personal high-level user-name and password. Access will be limited to the clinicians managing the patient and restricted members of the research team (with the patients' permission). A permanent audit trail of access will be kept.

Research data will be stored on secure, password-protected university computers with access limited to the named research team.

The only identifiable data variable sent via the Internet is the serial number of the blood pressure monitor, the glucometer and the scales.

8.2 Contamination between the intervention and control groups

The decision to randomise at the patient rather than the practice or service level avoids the methodological issues associated with cluster randomisation, but raises the possibility of contamination (i.e. the research also affecting the way patients in the control group are treated). This has been considered carefully and two potential forms of contamination identified.

Patients in both groups will have their care optimised at baseline, and will receive self-management education. Whilst this is recommended by guidelines as good care, it probably represents a higher standard than is usual.

In addition, practitioners may become more familiar with the concept of diabetes self-management and modify their management of patients in the control group. As most GPs will only have two or three patients in the trial, this is unlikely to affect their management of diabetes generally. Practice diabetic nurses are more likely to be influenced by the experience of working with patients in the intervention group. However, we anticipate that the key impact of the intervention will be on patients' engagement with their care as a result of the tele-monitoring rather than changing professional management.

These issues will be specifically explored in the qualitative interviews with professionals.

8.3 Home monitoring revealing problems which require intervention

It is possible that home monitoring may reveal something which, now it is known, is of concern. This could include very high blood pressure (systolic > 220mmHg) and extremes of blood glucose (<4mmol/l or >15mmol/l), low blood pressure causing symptoms or the patient being unable to record his/her blood pressure (possibly because of the onset of atrial fibrillation). The written information will tell the patient what to do in each instance and the patient will also have a laminated "ready reckoner" advising them what to do. Any adverse events will be recorded by clinical and study staff on an adverse events form (see Section 5.14).

8.4 Excess workloads for practices

It is envisaged that, when fully operational, the home monitoring service will not involve any more work for practices than the current system. Although some patients may require more attention, this will be offset by fewer patients needing to attend the surgery for routine blood pressure checks

8.5 Equipment Failures

There is no maintenance contract for the equipment loaned to the patient - non functioning blood pressure monitors and modems will be replaced from a small stock held by the trial manager. The functioning of the system will be audited by checking the online records of at least 10% of users against the stored values in their blood pressure monitors

9 Project Team and Task Allocation

- The project will be led by Prof Brian McKinstry with the support of Dr Sarah Wild, Dr John McKnight, Professor Paul Padfield and the Edinburgh Clinical Trials Unit.
- The trial will be managed by Trial Manager Mary Paterson with backup from Telescot programme manager Dr Lucy McCloughan
- The qualitative study will be led by Dr Janet Hanley who will line-manage the qualitative researcher, Mr Peter Fairbrother and project secretary who will be employed by Napier University.
- Dr Steff Lewis will supervise the statistical analysis
- Dr Marjon van der Pol will provide advice on the health economics analysis.

- Research nurses in Lothian will be employed through the Wellcome Trust Clinical Research Facilities in Edinburgh. Equivalent arrangements will be made in other areas.

10 Project Management and Quality Assurance

- The project team, consisting of grantholders and research staff, will meet at least monthly.
- The project will be overseen by the independent Trial Steering Committee for the TELESCOT programme which comprises the chief investigator; an independent chairperson; the applicants, trial staff and representatives of the funding body; and a patient representative (if possible). Three experienced trialists have agreed to oversee the individual RCTs for the telehealth programme of which this trial is a part; Professor Ann-Louise Kinmonth (diabetes), Professor Chris Griffiths (COPD trial) and Professor Lewis Ritchie (hypertension and stroke).
- The study will be carried out to GCP standards and managed within the Research Governance Framework. Ethical approval will be sought via the National Research Ethics Service and management approval from NHS Lothian.
- The Independent Trial Steering Committee will take on the role of a Data Monitoring & Ethics Committee. Adverse events will be analysed on a regular basis and where necessary the TSC will be empowered to terminate the study should there be any safety concerns to ensure the rights and well being of the trial participants. If examination of unblinded data is required to make a decision about the continuation of the study for any reason then an experienced independent trial statistician will be appointed to review the data and advise the committee.

11 Timescale

Timescale: Recruitment is anticipated to start in June 2011. Further details of the timescale are shown in the chart in Appendix 15.5.

12 Reporting

Six monthly progress reports and a final report will be provided to the funder in the format required. Reports will also be provided as required for the programme management group, steering group and data monitoring committee.

13 Finance

- The project is funded by an applied programme grant from the Chief Scientist Office. This award does not include university full economic costs;
- The funding is managed by NHS Lothian;
- A service level agreement has been developed between NHS Lothian and the University of Edinburgh for the clinical trial activity to be carried out by Edinburgh University;
- Support for science costs have been sought to recompense GP practices for time spent setting up this study including record searches and review and training;
- Service level agreements have been developed with the Wellcome Trust Clinical Research Facility, the Scottish Primary Care Research Network, and with practices outwith NHS Lothian as appropriate, to reflect their contribution to the study.

14 References

1. Auditor General for Scotland. *Managing Long Term Conditions*. 2007. Edinburgh, Audit Scotland.

2. Wanless, D. Securing our future health: Taking a long-term view. 2010. London, HM Treasury.

3. Scottish Executive. Delivering for Health. 2005. Edinburgh, Scottish Executive.

4. Gibson PG, Powell H, Coughlan J, Wilson AJ, Abramson M, Haywood P *et al*. Self-management education and regular practitioner review for adults with asthma. *Cochrane.Database.Syst.Rev.* 2003;CD001117.

5. Fahey T, Schroeder K, Ebrahim S. Interventions used to improve control of blood pressure in patients with hypertension. *Cochrane.Database.Syst.Rev.* 2006;CD005182.

6. Farmer A, Wade A, Goyder E, Yudkin P, French D, Craven A *et al*. Impact of self monitoring of blood glucose in the management of patients with non-insulin treated diabetes: open parallel group randomised trial. *BMJ* 2007;**335**:132.

7. Messerli FH, Williams B, Ritz E. Essential hypertension. *Lancet* 2007;**370**:591-603.

8. Peel E, Parry O, Douglas M, Lawton J. Blood glucose self-monitoring in non-insulin-treated type 2 diabetes: a qualitative study of patients' perspectives. *Br.J Gen.Pract.* 2004;**54**:183-8.

9. Glasziou P, Irwig L, Mant D. Monitoring in chronic disease: a rational approach. *BMJ* 2005;**330**:644-8.

10. Department of Health. Information for health: an information strategy for the modern NHS 1998-2005. 1998. London, Department of Health.

11. Department of Health. The Expert Patient: A new approach to chronic disease management for the 21st century. 2001. London, The Stationery Office.

12. Department of Health. Improving Chronic Disease Management. 2004. London, The Stationery Office.

13. Shea S, Weinstock RS, Starren J, Teresi J, Palmas W, Field L *et al*. A randomized trial comparing telemedicine case management with usual care in older, ethnically diverse, medically underserved patients with diabetes mellitus. *J Am Med.Inform.Assoc.* 2006;**13**:40-51.

14. McMahon GT, Gomes HE, Hickson HS, Hu TM, Levine BA, Conlin PR. Web-based care management in patients with poorly controlled diabetes. *Diabetes Care* 2005;**28**:1624-9.

15. Marrero DG, Vandagriff JL, Kronz K, Fineberg NS, Golden MP, Gray D *et al*. Using telecommunication technology to manage children with diabetes: the Computer-Linked Outpatient Clinic (CLOC) Study. *Diabetes Educ.* 1995;**21**:313-9.

16. Casas A, Troosters T, Garcia-Aymerich J, Roca J, Hernandez C, Alonso A *et al*. Integrated care prevents hospitalisations for exacerbations in COPD patients. *Eur.Respir.J* 2006;**28**:123-30.

17. Niesink A, de Weert, an Oene G, ., Schrijvers AJ. The effects of integrated care for COPD patients on quality of life. Review and research design. *Prim.Care Resp.J* 2006;**15**:2001.

18. Whitten PS, Mair FS, Haycox A, May CR, Williams TL, Hellmich S. Systematic review of cost effectiveness studies of telemedicine interventions. *BMJ* 2002;**324**:1434-7.

19. Medical Research Council. A framework for the development and evaluation of RCTs for complex interventions to improve health. 2000. London, Medical Research Council.

20. de Toledo P, Jimenez S, del Pozo F, Roca J, Alonso A, Hernandez C. Telemedicine experience for chronic care in COPD. *IEEE Trans.Inf.Technol.Biomed.* 2006;**10**:567-73.

21. Clark RA, Inglis SC, McAlister FA, Cleland JG, Stewart S. Telemonitoring or structured telephone support programmes for patients with chronic heart failure: systematic review and meta-analysis. *BMJ* 2007;**334**:942.

22. Pinnock H, Slack R, Pagliari C, Price D, Sheikh A. Understanding the potential role of mobile phone-based monitoring on asthma self-management: qualitative study. *Clin.Exp.Allergy* 2007;**37**:794-802.

23. Campbell NC, Murray E, Darbyshire J, Emery J, Farmer A, Griffiths F *et al*. Designing and evaluating complex interventions to improve health care. *BMJ* 2007;**334**:455-9.

24. Oakley A, Strange V, Bonell C, Allen E, Stephenson J. Process evaluation in randomised controlled trials of complex interventions. *BMJ* 2006;**332**:413-6.

25. Bonell C, Oakley A, Hargreaves J, Strange V, Rees R. Assessment of generalisability in trials of health interventions: suggested framework and systematic review. *BMJ* 2006;**333**:346-9.

26. Wild SH, Roglic G, Green A, Sicree R, King H. Global prevalence of diabetes: estimates for the year 2000 and projections for 2030. *Diabetes Care* 2004;**27**:1047-53.

27. Holman RR, Paul SK, Bethel MA, Neil HA, Matthews DR. Long-term follow-up after tight control of blood pressure in type 2 diabetes. *N.Engl.J Med.* 2008;**359**:1565-76.

28. Holman RR, Paul SK, Bethel MA, Matthews DR, Neil HA. 10-year follow-up of intensive glucose control in type 2 diabetes. *N.Engl.J Med.* 2008;**359**:1577-89.

29. Colhoun HM, Betteridge DJ, Durrington PN, Hitman GA, Neil HA, Livingstone SJ *et al*. Primary prevention of cardiovascular disease with atorvastatin in type 2 diabetes in the Collaborative Atorvastatin Diabetes Study (CARDS): multicentre randomised placebo-controlled trial. *Lancet* 2004;**364**:685-96.

30. Franklin VL, Greene A, Waller A, Greene SA, Pagliari C. Patients' engagement with "Sweet Talk" - a text messaging support system for young people with diabetes. *J Med.Internet.Res.* 2008;**10**:e20.

31. Little P, Barnett J, Barnsley L, Marjoram J, Fitzgerald-Barron A, Mant D. Comparison of agreement between different measures of blood pressure in primary care and daytime ambulatory blood pressure. *BMJ* 2002;**325**:254.

32. Pickering TG, Hall JE, Appel LJ, Falkner BE, Graves J, Hill MN *et al*. Recommendations for blood pressure measurement in humans and experimental animals: Part 1: blood pressure measurement in humans: a statement for professionals from the Subcommittee of Professional and Public Education of the American Heart Association Council on High Blood Pressure Research. *Hypertension* 2005;**45**:142-61.

33. ISD Scotland. Quality & Outcomes Framework 2007/08 Achievement and prevalence at Scotland and NHS Board level. http://www.isdscotland.org/ . 2008. 1-12-2009.

34. Chobanian AV, Bakris GL, Black HR, Cushman WC, Green LA, Izzo JL, Jr. *et al*. The Seventh Report of the Joint National Committee on Prevention, Detection, Evaluation, and Treatment of High Blood Pressure: the JNC 7 report. *JAMA* 2003;**289**:2560-72.

35. Scottish Executive Health Department. Delivering for Health. 2005. Edinburgh, Scottish Executive.

36. InMedica. The Market for Home-Use Digital Blood-Pressure Monitors 2006 - Worldwide. 2007. Austin, Texas, Intex Management Services.

37. Chumbler NR, Neugaard B, Kobb R, Ryan P, Qin H, Joo Y. Evaluation of a care coordination/home-telehealth program for veterans with diabetes: health services utilization and health-related quality of life. *Eval.Health Prof.* 2005;**28**:464-78.

38. National Institute for Health and Clinical Excellence. NICE guideline CG034: Management of hypertension in adults in Primary Care. 2006. London, NICE.

39. Verberk WJ, Kessels AG, de Leeuw PW. Prevalence, causes, and consequences of masked hypertension: a meta-analysis. *Am J Hypertens.* 2008;**21**:969-75.

40. Five-year findings of the hypertension detection and follow-up program. I. Reduction in mortality of persons with high blood pressure, including mild hypertension. Hypertension Detection and Follow-up Program Cooperative Group. *JAMA* 1979;**242**:2562-71.

41. Welschen LM, Bloemendal E, Nijpels G, Dekker JM, Heine RJ, Stalman WA *et al*. Self-monitoring of blood glucose in patients with type 2 diabetes who are not using insulin: a systematic review. *Diabetes Care* 2005;**28**:1510-7.

42. VanWormer JJ, Martinez AM, Benson GA, Crain AL, Martinson BC, Cosentino DL *et al*. Telephone counselling and home telemonitoring: the Weigh by Day Trial. *Am J Health Behav.* 2009;**33**:445-54.

43. O'Kane MJ, Bunting B, Copeland M, Coates VE. Efficacy of self monitoring of blood glucose in patients with newly diagnosed type 2 diabetes (ESMON study): randomised controlled trial. *BMJ* 2008;**336**:1174-7.

44. Pare G, Jaana M, Sicotte C. Systematic review of home telemonitoring for chronic diseases: the evidence base. *J Am Med.Inform.Assoc.* 2007;**14**:269-77.

45. Stevens RJ, Coleman RL, Adler AI, Stratton IM, Matthews DR, Holman RR. Risk factors for myocardial infarction case fatality and stroke case fatality in type 2 diabetes: UKPDS 66. *Diabetes Care* 2004;**27**:201-7.

46. Zigmond AS,.Snaith RP. The hospital anxiety and depression scale. *Acta Psychiatr.Scand.* 1983;**67**:361-70.

47. The Measurement and Valuation of Health Status Using EQ-5D: A European Perspective: Evidence from the EuroQol BIO MED Research Programme. Kluwer Academic Publishers, 2003.

48. Lorig KR, Sobel DS, Ritter PL, Laurent D, Hobbs M. Effect of a self-management program on patients with chronic disease. *Eff.Clin.Pract.* 2001;**4**:256-62.

49. Morisky DE, Green LW, Levine DM. Concurrent and predictive validity of a self-reported measure of medication adherence. *Med.Care* 1986;**24**:67-74.

50. Craig CL, Marshall AL, Sjostrom M, Bauman AE, Booth ML, Ainsworth BE *et al*. International physical activity questionnaire: 12-country reliability and validity. *Med.Sci.Sports Exerc.* 2003;**35**:1381-95.

51. Myers J, Bader D, Madhavan R, Froelicher V. Validation of a specific activity questionnaire to estimate exercise tolerance in patients referred for exercise testing. *Am Heart J* 2001;**142**:1041-6.

52. Fitzgerald JT, Funnell MM, Hess GE, Barr PA, Anderson RM, Hiss RG *et al*. The reliability and validity of a brief diabetes knowledge test. *Diabetes Care* 1998;**21**:706-10.

53. http://www.ons.gov.uk/census/2011-census/2011-census-questionnaire-content/question-and-content-recommendations-for-2011 Final recommended questions 2011 - Ethnic group 887.9 Kb Pdf document

54. McKinstry B, Hanley J, Heaney D, McCloughan L, Elton R, Webb DJ. Impact on hypertension control of a patient-held guideline: a randomised controlled trial. *Br.J Gen.Pract.* 2006;**56**:842-7.

55. O'Brien E, Mee F, Atkins N, O'Malley K. Accuracy of the SpaceLabs 90207 determined by the British Hypertension Society protocol. *J Hypertens.* 1991;**9**:573-4.

56. National Institute for Health and Clinical Excellence. NICE guideline CG66 Type 2 diabetes: the management of type 2 diabetes (update). 2008. London, NICE.

57. Curtis, L. and Netten, A. Unit Costs of Health and Social Care 2004. 2008. Personal Social Services Research Unit, University of Kent.

58. Stratton IM, Adler AI, Neil HA, Matthews DR, Manley SE, Cull CA *et al*. Association of glycaemia with macrovascular and microvascular complications of type 2 diabetes (UKPDS 35): prospective observational study. *BMJ* 2000;**321**:405-12.

59. Qualitative Research Practice: A Guide for Social Science Students and Researchers. London: Sage Publications, 2003.

60. Rogers E. Diffusion of Innovations. London: Macmillan, 2003.

61. Bandura A. Social learning theory. Englewood Cliffs, New Jersey: Prentice-Hall, 1977.

62. Bandura A. Social Foundations of Thought and Action – A Social Cognitive Theory. Englewood Cliffs, New Jersey: Prentice-Hall, 1986.

63. Williams F, Gibbons DV. Technology Transfer: A Communication Perspective. Newbury Park: Sage, 1990.

64. Mittman BS, Tonesk X, Jacobson PD. Implementing Clinical Practice Guidelines: Social Influence Strategies and Practitioner Behaviour Change. *Quality Review Bulletin* 1992;**18**:413-22.

15 Appendices

15.1 Model of telemetry supported self care

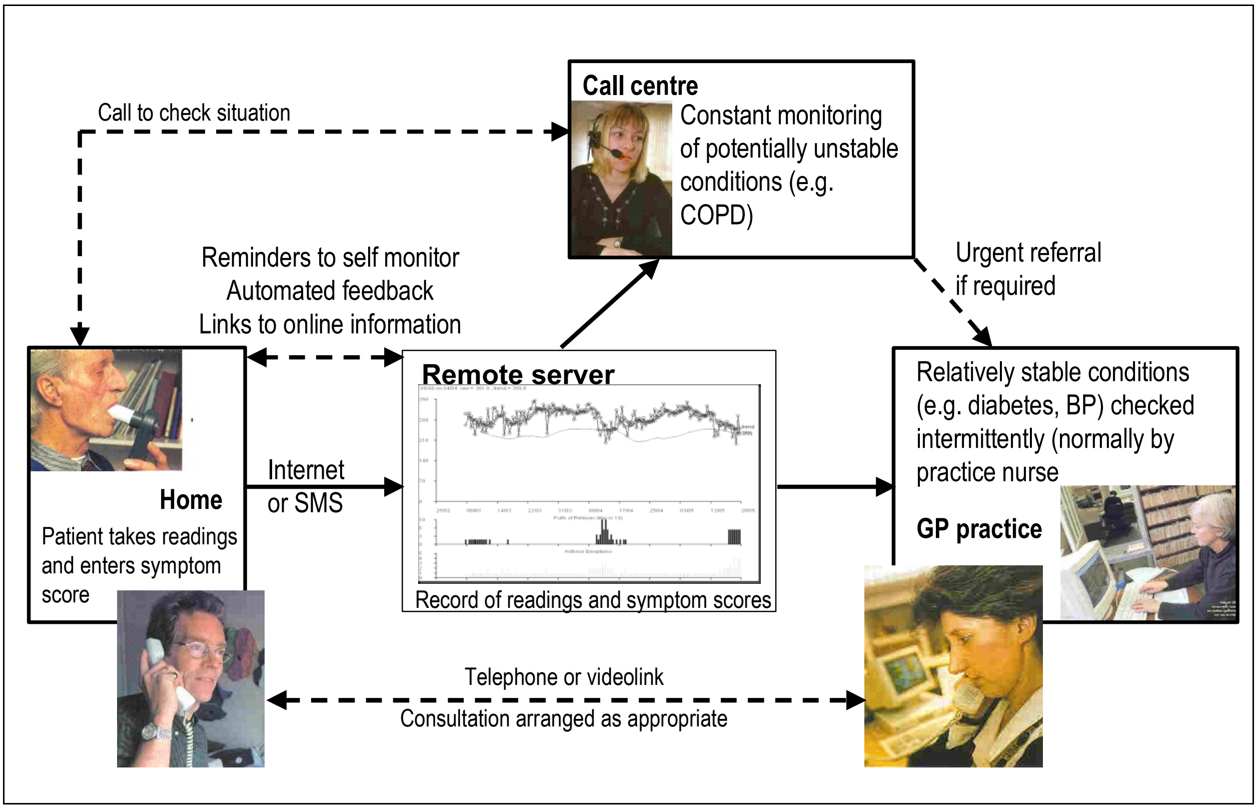

This model illustrates an overview of tele-monitoring. Two models are under consideration in our programme.

- In COPD (a potentially unstable condition) patients daily symptoms scores are scrutinised by a call-centre team who will contact the patient when responses to symptom questionnaire fall outside expected parameters or if readings are not transmitted. According to protocol, they will observed the following day, or referral made to a clinician who will be able to consult with the patient (either face-to-face or remotely via telephone or video-link) and arrange treatment as necessary.
- In the other models automatic responses to readings will be fed back to patients advising them if they need to contact their clinician (based on mean average of their results) or reminding them to take readings. Clinicians will view the patient record at regular intervals and contact patients by phone, email or text to give advice. Patients can also view results on line.

15.2 *Rapid Treatment Protocol. A Protocol for managing blood pressure in patients where home monitoring permits rapid escalation of treatment.*

This rapid treatment protocol is based on the Lothian Hypertension guidelines with the addition of timing suggestions and one change. The addition of a thiazide which is an *optional* first step in the Lothian Hypertension guideline (and also the NICE hypertension guideline) is delayed until step 3 because thiazides take some weeks to show an effect.

Treatment steps if control not achieved		
	**Step 1**	Calcium Channel Blocker or ACE inhibitor depending upon age ($\geq$ 50 CCB) – low dose
	↓	
After 2 weeks	*Dose titration*	*After 2 weeks dose titration of first drug (e.g. 5 to 10 mgs amlodipine or 10 to 20 mgs lisinopril)*
	↓	
After a further 2 weeks	**Step 2**	Calcium Channel Blocker and ACE inhibitor
	↓	
After a further 2 weeks	*Dose titration*	*After 2 weeks dose titration of second drug (e.g. 5 to 10 mgs amlodipine or 10 to 20 mgs lisinopril)*
	↓	
After a further 2 weeks	**Step 3**	Add a thiazide
	↓	
After a further 4 weeks (thiazides take longer to work)	**Step 4**	Add a ß blocker or spironolactone
	↓	
After a further 4 weeks	**Step 5**	Add whichever of step 4 had not been added

15.3 Type 2 Diabetes Blood-glucose-lowering therapy

HbA_{1c} ≥7.5 [1] after trial of lifestyle interventions

(A) Metformin [2]
1 500mg daily after main meal, increasing to 500mg twice daily after food; 2 850 mg twice daily so long as previous step did not cause diarrhoea; 3 If previous dose tolerated 850 mg three times daily after food. **Note** diarrhoea and abdominal symptoms may limit increases of metformin dose. My need to decrease dose if side effects and add steps from another drug. *See note on page 3.*

Consider sulfonylurea[4] here if:
- not overweight (tailor the assessment of body-weight-associated risk according to ethnic group[3]), or
- metformin is not tolerated or is contraindicated, or
- a rapid therapeutic response is required because of hyperglycaemic symptoms.

Fasting glucose X 2 ≥ 6 mmol/l [1]
Move to next treatment step

Fasting glucose X 2 < 6 mmol/l [1]
Monitor for deterioration

Metformin + sulfonylurea[4]
Maximum dose as per box (A) and (B)
See note on page 3

Consider substituting a DPP-4 inhibitor[9] or a thiazolidinedione[10] for the sulfonylurea if there is a significant risk of hypoglycaemia (or its consequences) or a sulfonylurea is contraindicated or not tolerated.

Fasting glucose X 2 ≥ 6 mmol/l [1]

Fasting glucose X 2 < 6 mmol/l [1]
Monitor for deterioration

Add insulin [2, 8], particularly if the person is markedly hyperglycaemic.
Insulin glargine started according to treat to target protocol.
Insulin + metformin + sulfonylurea [4]

Consider adding sitagliptin or a thiazolidinedione[10] instead of insulin if insulin is unacceptable (because of employment, social, recreational or other personal issues, or obesity).
Consider adding exenatide[6] (1 5 ug twice daily increasing after one month to 10 ug twice daily. Must be given by injection. System to start this will have to be set up to support the study) to metformin and a sulfonylurea if:

- BMI ≥ 35 kg/m2 in people of European descent[7] and there are problems associated with high weight, or
- BMI < 35 kg/m2 and insulin is unacceptable because of occupational implications or weight loss would benefit other comorbidities.

Fasting glucose X 2 ≥ 6 mmol/l [1]

Fasting glucose X 2 < 6 mmol/l [1]
Monitor for deterioration

Increase insulin dose and intensify regimen over time.
Consider pioglitazone (1 15 mg daily; 2 30 mg daily; 3 45 mg daily. Contraindicated in heart failure. May cause oedema. Clinical effect may be gradual so consider trends when considering next step. Patient must be warned to consult urgently if becoming breathless and also to seek advice if develops oedema) with insulin if:
- a thiazolidinedione has previously had a marked glucose-lowering effect, or
- blood glucose control is inadequate with high-dose insulin.

---

1 Or individually agreed target.
2 With active dose titration.
3 See the NICE clinical guideline on obesity (www.nice.org.uk/CG43).
4 Offer once-daily sulfonylurea if adherence is a problem.
5 Only continue sitagliptin or thiazolidinedione if reduction in HbA_{1c} of at least 0.5 percentage points in 6 months.
6 Only continue exenatide if reduction in HbA_{1c} of at least 1 percentage point and weight loss of at least 3% of initial body weight at 6 months.

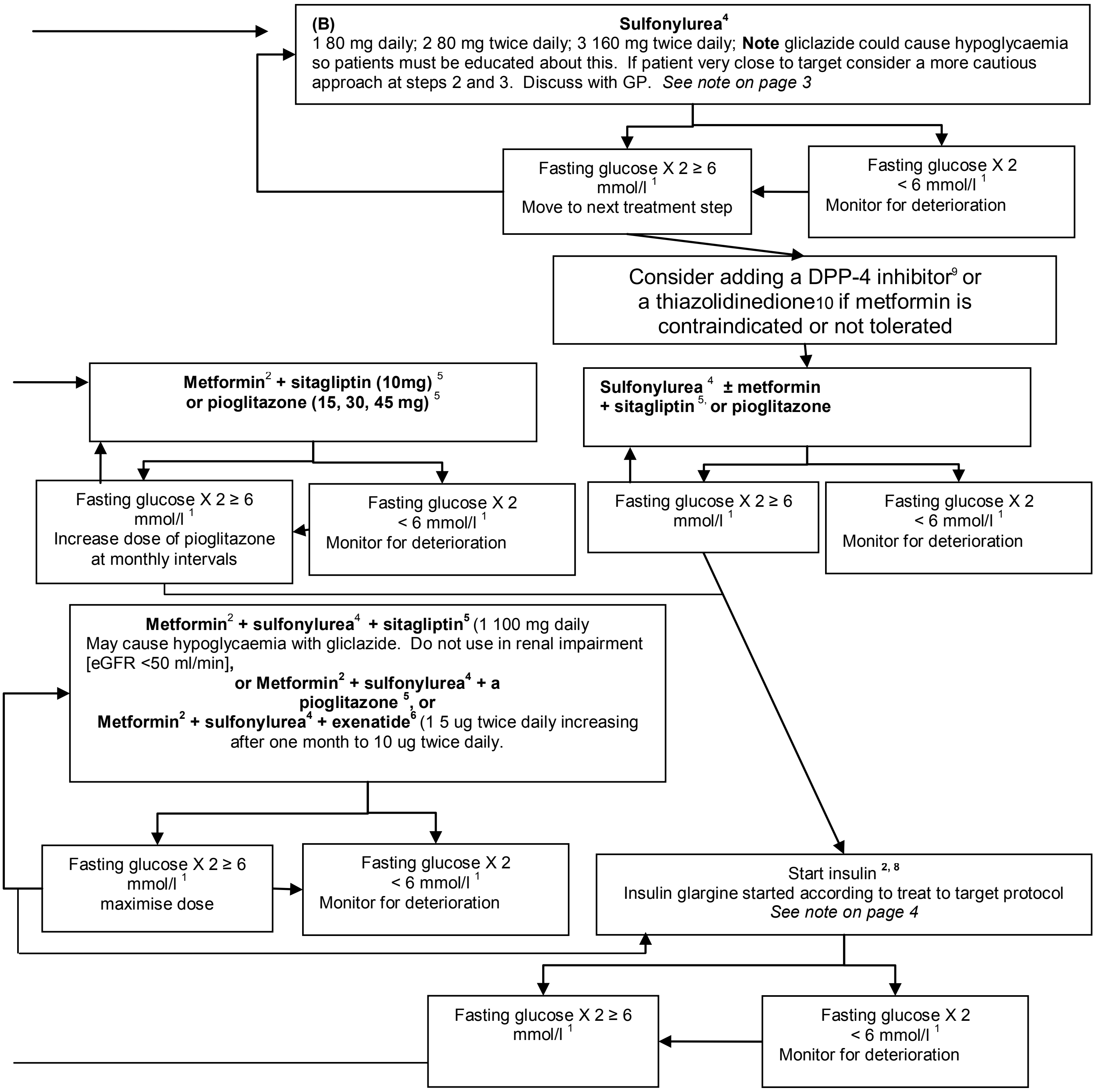

7 With adjustment for other ethnic groups.

8 Continue with metformin and sulfonylurea (and acarbose, if used), but only continue other drugs that are licensed for use with insulin. Review the use of sulfonylurea if hypoglycaemia occurs.

Metformin

● Step up metformin over several weeks to minimise risk of gastrointestinal (GI) side effects.
● Consider trial of extended-absorption metformin if GI tolerability prevents the person continuing with metformin.
● Review metformin dose if serum creatinine > 130 μmol/litre or estimated glomerular filtration rate (eGFR) < 45 ml/minute/1.73-m2.
● Stop metformin if serum creatinine > 150 μmol/litre or the eGFR < 30 ml/minute/1.73-m2.
● Prescribe metformin with caution for those at risk of a sudden deterioration in kidney function, and those at risk of eGFR falling to< 45 ml/minute/1.73-m2.
● If the person has mild to moderate liver dysfunction or cardiac impairment, discuss benefits of metformin so due consideration can be given to its cardiovascular-protective effects before any decision is made to reduce the dose.

Sulfonylureas

● Prescribe a sulfonylurea with a low acquisition cost (not glibenclamide) when an insulin secretagogue is indicated.
● Educate the person about the risk of hypoglycaemia, particularly if he or she has renal impairment.

Sitagliptin

● Continue Sitagliptin therapy only if there is a reduction of ≥ 0.5 percentage points in HbA1c in 6 months.
● Discuss the benefits and risks of Sitagliptin with the person, bearing in mind that Sitagliptin might be preferable to a Pioglitazone if:
- further weight gain would cause significant problems, or
  - A Pioglitazone is contraindicated, or
  - the person had a poor response to or did not tolerate a Pioglitazone in the past.

Pioglitazone

● Continue Pioglitazone therapy only if there is a reduction of ≥ 0.5 percentage points in HbA1c in 6 months.
● Discuss the benefits and risks of a Pioglitazone with the person, bearing in mind that a Pioglitazone might be preferable to Sitagliptin if:
- the person has marked insulin insensitivity, or
- Sitagliptin is contraindicated, or
- the person had a poor response to or did not tolerate Sitagliptin in the past.

● Do not start or continue a Pioglitazone if the person has heart failure or is at higher risk of fracture.
● When selecting Pioglitazone, take into account the most up-to-date advice from regulatory authorities, cost, safety and prescribing issues.

Exenatide

● Continue exenatide only if the person has a reduction in HbA1c of ≥ 1.0 percentage point and ≥ 3% of initial body weight in 6 months.
● Discuss the benefits of exenatide to allow the person to make an informed decision.

Acarbose

● Consider acarbose for a person unable to use other oral glucose-lowering medications.

Starting insulin therapy

● If other measures do not keep HbA1c to < 7.5% (or other agreed target), discuss benefits and risks of insulin treatment.
● Initiate with a structured programme.
● Begin with human NPH insulin taken at bedtime or twice daily according to need.
● Alternatively, consider a once-daily long-acting insulin analogue (insulin detemir, insulin glargine) if:
- the person needs help with injecting insulin and a long-acting insulin analogue would reduce injections from twice to once daily, or
- the person's lifestyle is restricted by recurrent symptomatic hypoglycaemic episodes, or
- the person would otherwise need twice-daily basal insulin injections plus oral glucose lowering drugs, or
- the person cannot use the device to inject NPH insulin.
● Consider twice-daily biphasic human insulin (pre-mixed) (particularly if HbA1c ≥9.0%). A once-daily regimen may be an option.
● Consider pre-mixed preparations of insulin analogues (including short-acting insulin analogues) rather than pre-mixed human insulin preparations if:
- immediate injection before a meal is preferred, or
- hypoglycaemia is a problem, or
- blood glucose levels rise markedly after meals.
● Consider switching to a long-acting insulin analogue (insulin detemir, insulin glargine) from NPH insulin if the person:
- does not reach target HbA1c because of hypoglycaemia, or
- has significant hypoglycaemia with NPH insulin irrespective of HbA1c level, or
- cannot use the delivery device for NPH insulin but could administer a long-acting insulin analogue, or
- needs help to inject insulin and could reduce the number of injections with a long-acting analogue.
● Review use of sulfonylurea if hypoglycaemia occurs with insulin plus sulfonylurea.

Intensifying the insulin regimen

● Monitor those using basal insulin regimens (NPH or a long-acting analogue [insulin detemir, insulin glargine]) for need for short-acting insulin before meals or pre-mixed insulin.
● Monitor those using pre-mixed insulin once or twice daily for need for further injection of short-acting insulin before meals or change to mealtime plus basal regimen.

Insulin delivery devices

● Offer education to a person who requires insulin about using an injection device (usually a pen injector and cartridge or a disposable pen) that they and/or their carer find easy to use.
● If a person has a manual or visual disability and requires insulin, offer an appropriate device or adaptation that can be used successfully.
● Appropriate local arrangements should be in place for the disposal of sharps.

15.4 Theoretical framework for telehealth studies

The technologies being trialled in this programme enable frequent self monitoring, organisation of the resultant data, and sharing of the data between patients and healthcare professionals in a way which has not been previously available in Primary Care. This is a complex intervention involving the following elements:

- Self monitoring. Although everyone monitors their own health, for most of the patients involved in this trial, formal measurement of symptoms and vital signs will be new to them as it was previously the responsibility of healthcare professionals. The exceptions will be blood glucose measurement for the diabetic patients and blood pressure monitoring for the 30% of patients that our pilot work suggests already own their own home monitors.
- The information itself. In most cases the parameters of interest have not been routinely and frequently monitored so the data, in conjunction with the way in which it is organised and shared, provides novel information about the condition and its control to the patients and their healthcare professionals.
- The organisation of the data. The systems used in these trials are able to organise and display the data provided in different ways e.g. an average blood pressure. They also provide some interpretation of the data based on pre-determined parameters.
- Automated feedback and reminders. The systems used in this trial are able to provide timely reminders and automated feedback for patients, which will not have been previously available to them
- The way in which the data are shared and used. The data record will be almost instantly available to both patients and healthcare professionals.

It is hypothesised that tele-monitoring will impact at three levels:

1. Patients may improve their knowledge and understanding of their condition as they monitor and receive timely and relevant feedback on their situation.
2. Professionals may feel more confident to offer self-management within the structure and on-going supervision of tele-monitoring
3. Appropriate access to services may be facilitated by the tele-monitoring as patients become more confident when they need help and advice and with the easy sharing of monitoring data.

The theoretical model developed by Glasziou *et al.* describes the complementary and evolving roles of periodic professional reviews and on-going patient self-monitoring.[1] A newly diagnosed condition is assessed and brought under control with professional support, before the patient assumes responsibility for self-management as the stable maintenance phase is established.

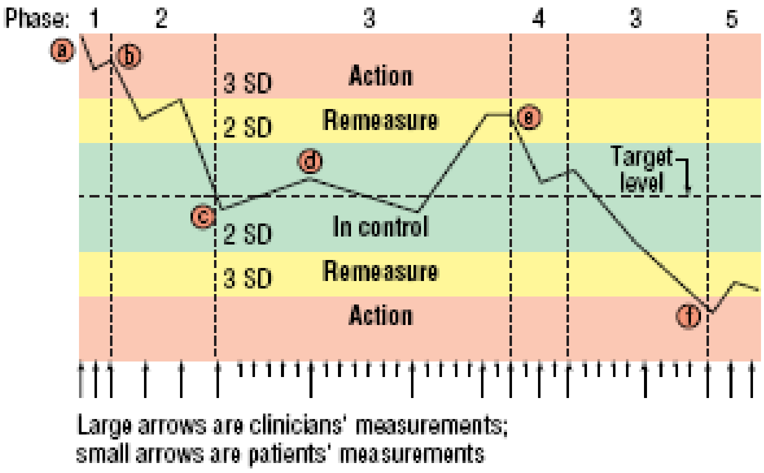

Figure 1. The five phases of treatment monitoring. After Glaziou *et al.*[1]

The context for the tele-monitoring trials is the maintenance phase where patients are monitoring a relatively stable situation, and expected to act (either by initiating treatment, or

seeking appropriate professional advice) if measurements fall outside pre-defined limits. The impact of this will vary according to the condition being monitored. For people with an asymptomatic condition such as hypertension this may the first time they have been aware of their control of a daily basis. Patients with COPD often have difficulty distinguishing the onset of an exacerbation from a 'bad day',[2, 3] and one possible mode of action is that tele-monitoring will define exacerbations more clearly and increase patient's confidence to commence treatment promptly.

Kennedy argues for a whole systems approach to the provision of effective support for self-care.[4]

Figure 2. Example of an intervention based on a whole systems perspective. After Kennedy *et al*.[4]

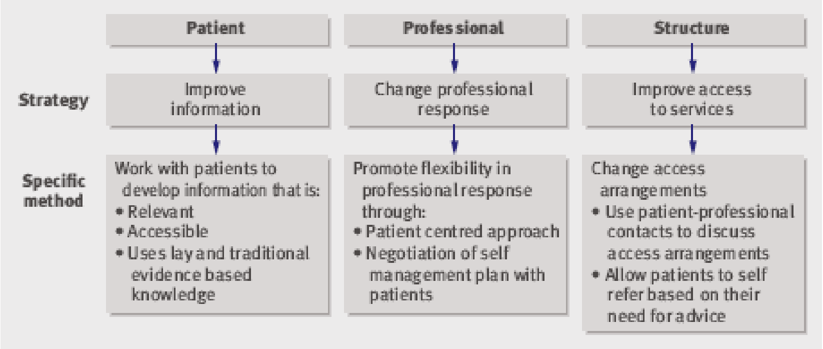

The anticipated outcomes are dependent on changes to behaviour: i.e. whether patients and/or professionals successfully use the equipment and change the way they manage the long term conditions being monitored. However, the way people act when they come into contact with an innovation does not always appear logical. There is a large literature on both the diffusion of innovations literature and health behaviour change, both underpinned by social learning theory which suggests people's perceptions of a situation determine how they behave.[5] Factors likely to influence this include

- self-efficacy, or belief in one's ability to perform a behaviour.[6]
- Perceived benefits weighed against perceived barriers to the action.[7,8]
- Perceptions of the attitudes of important others to the behaviour.[6,9]
- Reinvention and identity.[610]

The 'diffusion of innovations' model is the most comprehensive description of how new technologies (including behaviours) are adopted.[6] It includes a pattern of how an innovation spreads through a social group, a comparison of characteristics of those who adopt the innovation at different stages of its spread, and a staged model of the innovation-decision process (knowledge, persuasion, decision, implementation, confirmation), which an individual goes through when deciding to adopt or reject an innovation. Factors which may influence this process include the mode of communication (e.g. the presence of a change agent), prior conditions (including previous practice, felt needs and problems, innovativeness and norms of the social system), perceived characteristics of the innovation, disruptive or competing technologies or path dependence which may lock other technologies in place. A (self) criticism of this model is that it does not explain why individuals adopt or reject particular innovations at an early stage, sometimes seemingly irrationally.[6]

A related, but more explanatory model, the 'technology transfer communication and feedback' model[7] suggests that much of the unpredictability in the adoption of new technologies arises because individuals do not share a common perception of it or their need for it.[7] It introduces the concepts of technology push (the perceived merits of the new technology), and market pull (the perceived need for the new technology), both being required for the successful transfer of the new technology into practice.

Because of the complexity of the intervention, the explanatory aspect of this programme will be qualitative and involve interviews, focus groups and observation with patients, professionals, carers, and service planners. It will explore prior perceptions and conditions, the experience of using the system, and changes to perceptions and behaviour. The initial topic guides and data coding frame will be based on the factors identified by the diffusion of

innovations, social learning theory and our pilot work, but will be developed iteratively and be open to new approaches. The exceptions to the qualitative approach will be that patients will be asked to complete a quantitative measure of self-efficacy in chronic disease,[11] and (in the case of COPD) an assessment of knowledge about the respiratory condition.[12] The 'Self-Efficacy for Managing Chronic Disease' scale is widely used and will give some comparability with other research.

A specific aim of the qualitative work will be to explore the apparent paradox or tension in this service model where the aim is to increase self-care, but the model also increases professional surveillance of the patient. Our qualitative pilot work with hypertensive patients showed that even patients who were very committed to self-management welcomed this, but was not detailed enough to explain why. Work in the fields such as asthma monitoring and obesity management have also highlighted this paradox – that interventions which apparently take some control away from patients can result in their feeling of overall control increasing.[13, 14] These are important issues in a policy context which advocates increasing self-care.

1. Glasziou P, Irwig L, Mant D. Monitoring in chronic disease: a rational approach. *BMJ* 2005;330:644-8
2. Adams R, Chavannes N, Jones K, Subbe-Ostergaard M, Price D Exacerbations of chronic obstructive pulmonary disease - a patients' perspective *Prim Care Respir J* 2006; 15: 102-9
3. Calverley P, Pauwels R, Lofdahl G-G, Svensson K, Higenbottam T, Carlsson L-G, Stahl E. Relationship between respiratory symptoms and medical treatment in exacerbations of COPD. *Eur Respir J* 2005; 26: 406-413
4. Kennedy A, Rogers A, Bower P. Support for self care for patients with chronic disease. *BMJ* 2007; 335: 968-970
5. Bandura A (1977) *Social Learning Theory*, Prentice-Hall, Englewood Cliffs, New Jersey
6. Bandura A (1986) *Social Foundations of Thought and Action – A Social Cognitive Theory.* Prentice-Hall, Englewood Cliffs, New Jersey
7. Rogers E (2003). *Diffusion of Innovations*, 5th Edition, Macmillan, London
8. Williams F, Gibson DV. *Technology Transfer: A Communication Perspective*. Sage, Newbury Park 1990
9. Mittman BS, Tonesk X, Jacobson PD. Implementing Clinical Practice Guidelines: Social Influence Strategies and Practitioner Behaviour Change. *Quality Rev Bull* 1992; 18: 413-422
10. Ogden J, *Seeing the light: sustained behaviour change and the process of reinvention*. The British Psychological Society 2007. http://www.dhp2007.org.uk/document-download-area/document-download$.cfm?file_uuid=F9AEFFB0-1143-DFD0-7EA4-7FB84D95F79C&ext=doc
11. Stanford Patient Education Research Center. *Self-Efficacy for Managing Chronic Disease 6-Item Scale.* http://patienteducation.stanford.edu/research/secd6.html
12. Hyland M, Jones R, Hanney K. The Lung Information Needs Questionnaire: Development, preliminary validation and findings. *Respir Med* 2006; 100: 1807-1816
13. Pinnock H, Slack R, Pagliari C, Price D, Sheikh A. Understanding the potential role of mobile phone based monitoring on asthma self-management: qualitative study. *Clin Exp Allergy* 2007*; 3: 794–802*
14. Ogden, J., Clementi, C., Aylwin, S., Patel, A. (2005) Exploring the impact of obesity surgery on patient's health status: a quantitative and qualitative study. *Obesity Surgery;* 15: 266-272.

15.5 Timescales

	Sep-10	Oct 10	Nov-10	Dec-10	Jan-11	Feb-11	Mar-11	Apr-11	May-11	Jun-11	Jul-11	Aug-11	Sep-11	Oct-11	Nov-11	Dec-11	Jan-12	Feb-12	Mar-12	Apr-12	May-12	Jun-12	Jul-12	Aug-12	Sep-12	Oct-12	Nov-12	Dec-12	Jan-13	end
**Diabetes (CSO)**																														
develop protocol	■	■																												
source devices	■	■	■																											
produce study paperwork	■																													
Ethics & R&D	■	■																												
TSC ratification	■																													
practice recruitment strategy		■	■	■																										
database development	■	■	■	■																										
finalise devices equipment	■	■																												
adoption by SPCRN & SDRN	■	■																												
complete TMF & ISF		■																												
nurse training			■	■																										
print up pilot materials				■																										
print up final materials							■																							
pilot study in Blackburn					■	■	■																							
recruit practices					■	■	■																							
recruit patients							■	■	■	■	■	■	■	■	■															
baseline measures								■	■	■	■	■	■	■	■	■														
qualitative work																														
endpoint measures																	■	■	■	■	■	■	■	■	■					
database lock																										■				
health economic analysis																											■	■	■	
data analysis																											■	■	■	
report writing																									■	■	■	■	■	
