# Supplementary material for: Supported Telemonitoring and Glycemic Control in People with Type 2 Diabetes: The Telescot Diabetes Pragmatic Multicenter Randomized Controlled Trial
Source: PLoS Med. 2016 Jul 26;13(7):e1002098. doi: 10.1371/journal.pmed.1002098 (PMC4961438; doi:10.1371/journal.pmed.1002098)
Supplement: S3 Text — (DOCX) [file pmed.1002098.s013.docx]

**S3 Text: Supplementary information about the intervention for the Telescot diabetes pragmatic randomized controlled trial**

The family practices and participants were asked to use a system that comprised of a validated glucose meter, an electronic home blood pressure monitor and weighing scales, with a modem. The modem enabled the transfer of blood glucose, BP and weight readings via SMS to a secure website that was accessible to the users and their healthcare team. The monitors linked to the modem, via Bluetooth.

The intervention comprised several components:

*Home blood glucose monitoring*—participants were asked to measure their blood glucose morning and evening twice weekly. . Some patients were included who were already on insulin and using glucose meters daily at the start of the trial. Their readings were recorded more frequently as required. Participants who required the addition of insulin to their treatment during the course of the trial could continue to use this system and also recorded more frequent readings.

*Home blood pressure monitoring*— participants were asked to check their blood pressure 10-20 times over the first seven days to establish a reliable average and then weekly if their average blood pressure was within the recommended range. If, however, they had made any change to lifestyle or drugs that would impact on their blood pressure, they were asked to measure their blood pressure for a more intensive period of monitoring to allow the rolling average to change and more quickly assess the effect. The guidance was not restrictive.

*Home weight monitoring*-the participants were asked to weigh themselves weekly in the morning

*Transmission of data*—this simply required the modem to be switched on and to have a signal when the measurements were taken. Participants measured their blood sugar using a lancet and strip with the glucose meter and the reading from the glucose meter was sent via a glucose meter accessory plugged into the base of the monitor. They had to apply the cuff and press a button on the blood pressure monitor and they had to step onto the weighing scales to send their weight measurement. The transmission of the readings occurred automatically with all three monitors.

*Feedback to patient participants-*the user could securely access their record on the server at any time via the Internet and could also opt to receive monthly reports via email. These reports displayed readings in tabular and graphical form and included the average blood pressure and the average blood glucose reading before 10.00am and after 10.00am.

*Sharing readings with the family practice team*—members of the doctors and nurses in the family practicem were able to access the records of their patients online via a secure login to a summary screen, which listed their patients, their average blood glucose, blood pressure and weight over the last week, and the date of their last readings. Patients with high readings were highlighted by the software for easy identification. Clicking on the name of individual patients led to lists or graphs of all their readings. The doctor or nurse could then check their patients’ electronic healthcare (family practice) record to see if there had been recent advice about drug or lifestyle change and, if not, could contact the patient to make a change. Treatment for diabetes could be altered according to the protocol every 4 weeks with the aim of maintaining consecutive fasting blood levels between 4 and 6mmol/l. Anti-hypertensive drug treatment could be altered every 4-6 weeks until the mean blood pressure based on the last 10 readings was at or below the target of 130mmHg. Doctors and nurses were recommended to check the website weekly, but they could choose the frequency of log on.

The user was also given contact details and encouraged to make contact by email or by telephoning to discuss their treatment if their blood glucose remained uncontrolled or their blood pressure remained high. In the event of a very high blood pressure (>220 mmHg) or very high blood glucose levels (>15mmol/l) patients were asked to repeat the measurements and if they remained high patients were be advised to contact their doctor or nurse urgently for advice. Patients recording very low blood glucose (<4mmol/l) were advised to eat or take a glucose drink and, if hypoglycaemia persisted, to contact their doctor or nurse

Within the family practice, no additional infrastructure was required, but primary care nurses needed to set aside some time to answer telephone / email queries from patients, and required access to the records on the remote server.

*Usual care*

Participants allocated to the usual care group were asked to continue to attend their family practice for diabetes and blood pressure checks according to their usual routine. (normally at least twice a year). If they were already monitoring their blood sugar or blood pressure at home they were not discouraged.

*All participants*

All participants were given an information pack containing a range of publicly available leaflets on the management of diabetes and lifestyle modification.
