# Supplementary material for: Supported Telemonitoring and Glycemic Control in People with Type 2 Diabetes: The Telescot Diabetes Pragmatic Multicenter Randomized Controlled Trial
Source: PLoS Med. 2016 Jul 26;13(7):e1002098. doi: 10.1371/journal.pmed.1002098 (PMC4961438; doi:10.1371/journal.pmed.1002098)
Supplement: S4 Text — (DOCX) [file pmed.1002098.s014.docx]

**S3 Text: Treatment algorithms for blood pressure and diabetes for the Telescot diabetes pragmatic randomized controlled trial**

Algorithms for blood pressure and glucose management as given in Appendix 15.2 and Appendix 15.3 of the trial protocol respectively.

## Rapid Treatment Protocol. A Protocol for managing blood pressure in patients where home monitoring permits rapid escalation of treatment.

This rapid treatment protocol is based on the Lothian Hypertension guidelines with the addition of timing suggestions and one change. The addition of a thiazide which is an *optional* first step in the Lothian Hypertension guideline (and also the NICE hypertension guideline) is delayed until step 3 because thiazides take some weeks to show an effect.

| Treatment steps if control not achieved |  |  |
| --- | --- | --- |
|  | **Step 1** | Calcium Channel Blocker or ACE inhibitor depending upon age (> 50 CCB) – low dose |
|  | 🢃 |  |
| After 2 weeks | *Dose titration* | *After 2 weeks dose titration of first drug (e.g. 5 to 10 mgs amlodipine or 10 to 20 mgs lisinopril)* |
|  | 🢃 |  |
| After a further 2 weeks | **Step 2** | Calcium Channel Blocker and ACE inhibitor |
|  | 🢃 |  |
| After a further 2 weeks | *Dose titration* | *After 2 weeks dose titration of second drug (e.g. 5 to 10 mgs amlodipine or 10 to 20 mgs lisinopril)* |
|  | 🢃 |  |
| After a further 2 weeks | **Step 3** | Add a thiazide |
|  | 🢃 |  |
| After a further 4 weeks (thiazides take longer to work) | **Step 4** | Add a ß blocker or spironolactone |
|  | 🢃 |  |
| After a further 4 weeks | **Step 5** | Add whichever of step 4 had not been added |

## Type 2 Diabetes Blood-glucose-lowering therapy

HbA_1c_ ≥7.5 ^1^ after trial of lifestyle interventions

**(A) Metformin ^2^**

1 500mg daily after main meal, increasing to 500mg twice daily after food; 2 850 mg twice daily so long as previous step did not cause diarrhoea; 3 If previous dose tolerated 850 mg three times daily after food. **Note** diarrhoea and abdominal symptoms may limit increases of metformin dose. My need to decrease dose if side effects and add steps from another drug. *See note on page 3*.

Fasting glucose X 2 ≥ 6 mmol/l ^1^

Move to next treatment step

Fasting glucose X 2

< 6 mmol/l ^1^

Monitor for deterioration

**Metformin + sulfonylurea^4^**

Maximum dose as per box (A) and (B)

*See note on page 3*

Add insulin ^2, 8^, particularly if the person is

markedly hyperglycaemic.

Insulin glargine started according to treat to target protocol.

**Insulin + metformin + sulfonylurea ^4^**

Consider sulfonylurea^4^ here if:

● not overweight (tailor the assessment

of body-weight-associated risk according to ethnic group^3^), or

● metformin is not tolerated or is contraindicated, or

● a rapid therapeutic response is required because of hyperglycaemic symptoms.

Consider substituting a DPP-4 inhibitor^9^ or a thiazolidinedione^10^ for the sulfonylurea if there is a significant risk of hypoglycaemia (or its consequences) or a sulfonylurea is contraindicated or not tolerated.

Consider adding sitagliptin or a thiazolidinedione^10^ instead of insulin if insulin is unacceptable (because of employment, social, recreational or other personal issues, or obesity).

Consider adding exenatide^6^ (1 5 ug twice daily increasing after one month to 10 ug twice daily. Must be given by injection. System to start this will have to be set up to support the study) to metformin and a sulfonylurea if:

• BMI ≥ 35 kg/m2 in people of European descent^7^ and there are problems associated with high weight, or

• BMI < 35 kg/m2 and insulin is

unacceptable because of occupational implications or weight loss would benefit other comorbidities.

Increase insulin dose and intensify regimen over time.

Consider pioglitazone (1 15 mg daily; 2 30 mg daily; 3 45 mg daily. Contraindicated in heart failure. May cause oedema. Clinical effect may be gradual so consider trends when considering next step. Patient must be warned to consult urgently if becoming breathless and also to seek advice if develops oedema) with insulin if:

• a thiazolidinedione has previously

had a marked glucose-lowering

effect, or

• blood glucose control is inadequate

with high-dose insulin.

Fasting glucose X 2 ≥ 6 mmol/l ^1^

Fasting glucose X 2 ≥ 6 mmol/l ^1^

Fasting glucose X 2

< 6 mmol/l ^1^

Monitor for deterioration

Fasting glucose X 2

< 6 mmol/l ^1^

Monitor for deterioration

1 Or individually agreed target.

2 With active dose titration.

3 See the NICE clinical guideline on obesity (www.nice.org.uk/CG43).

4 Offer once-daily sulfonylurea if adherence is a problem.

5 Only continue sitagliptin or thiazolidinedione if reduction in HbA1c

of at least 0.5 percentage points in 6 months.

6 Only continue exenatide if reduction in HbA1c of at least 1 percentage

point and weight loss of at least 3% of initial body weight at 6 months.

**(B) Sulfonylurea^4^**

1 80 mg daily; 2 80 mg twice daily; 3 160 mg twice daily; **Note** gliclazide could cause hypoglycaemia so patients must be educated about this. If patient very close to target consider a more cautious approach at steps 2 and 3. Discuss with GP. *See note on page 3*

Consider adding a DPP-4 inhibitor^9^ or

a thiazolidinedione10 if metformin is contraindicated or not tolerated

**Sulfonylurea** ^4^ **± metformin**

**+ sitagliptin** ^5,^ **or pioglitazone**

**Metformin**^2^ **+ sitagliptin (10mg)** ^5^

**or pioglitazone (15, 30, 45 mg)** ^5^

**Metformin**^2^ **+ sulfonylurea**^4^  **+ sitagliptin^5^** (1 100 mg daily

May cause hypoglycaemia with gliclazide. Do not use in renal impairment [eGFR <50 ml/min]**,**

**or Metformin^2^ + sulfonylurea^4^ + a**

**pioglitazone ^5^, or**

**Metformin^2^ + sulfonylurea^4^ + exenatide^6^** (1 5 ug twice daily increasing after one month to 10 ug twice daily.

Must be given by injection. System to start this will have to be set up to support the study.

)

Start insulin **^2, 8^**

Insulin glargine started according to treat to target protocol

*See note on page 4*

7 With adjustment for other ethnic groups.

8 Continue with metformin and sulfonylurea (and acarbose, if used), but only continue other drugs that are licensed for use with insulin. Review the use of sulfonylurea if hypoglycaemia occurs.

Fasting glucose X 2 ≥ 6 mmol/l ^1^

Move to next treatment step

Fasting glucose X 2 ≥ 6 mmol/l ^1^

Increase dose of pioglitazone at monthly intervals

Fasting glucose X 2 ≥ 6 mmol/l ^1^

Fasting glucose X 2 ≥ 6 mmol/l ^1^

maximise dose

Fasting glucose X 2 ≥ 6 mmol/l ^1^

Fasting glucose X 2

< 6 mmol/l ^1^

Monitor for deterioration

Fasting glucose X 2

< 6 mmol/l ^1^

Monitor for deterioration

Fasting glucose X 2

< 6 mmol/l ^1^

Monitor for deterioration

Fasting glucose X 2

< 6 mmol/l ^1^

Monitor for deterioration

Fasting glucose X 2

< 6 mmol/l ^1^

Monitor for deterioration

**Metformin**

● Step up metformin over several weeks to minimise risk of gastrointestinal (GI) side effects.

● Consider trial of extended-absorption metformin if GI tolerability prevents the person

continuing with metformin.

● Review metformin dose if serum creatinine > 130 μmol/litre or estimated glomerular

filtration rate (eGFR) < 45 ml/minute/1.73-m2.

● Stop metformin if serum creatinine > 150 μmol/litre or the eGFR < 30 ml/minute/1.73-m2.

● Prescribe metformin with caution for those at risk of a sudden deterioration in kidney

function, and those at risk of eGFR falling to< 45 ml/minute/1.73-m2.

● If the person has mild to moderate liver dysfunction or cardiac impairment, discuss

benefits of metformin so due consideration can be given to its cardiovascular-protective effects

before any decision is made to reduce the dose.

**Sulfonylureas**

● Prescribe a sulfonylurea with a low acquisition cost (not glibenclamide) when an insulin

secretagogue is indicated.

● Educate the person about the risk of hypoglycaemia, particularly if he or she has

renal impairment.

**Sitagliptin**

● Continue Sitagliptin therapy only if there is a reduction of ≥ 0.5 percentage points in HbA1c

in 6 months.

● Discuss the benefits and risks of Sitagliptin with the person, bearing in mind that

Sitagliptin might be preferable to a Pioglitazone if:

– further weight gain would cause significant problems, or

- A Pioglitazone is contraindicated, or
- the person had a poor response to or did not tolerate a Pioglitazone in the past.

**Pioglitazone**

● Continue Pioglitazone therapy only if there is a reduction of ≥ 0.5 percentage points in

HbA1c in 6 months.

● Discuss the benefits and risks of a Pioglitazone with the person, bearing in

mind that a Pioglitazone might be preferable to Sitagliptin if:

– the person has marked insulin insensitivity, or

– Sitagliptin is contraindicated, or

– the person had a poor response to or did

not tolerate Sitagliptin in the past.

● Do not start or continue a Pioglitazone if the person has heart failure or is at higher risk of

fracture.

● When selecting Pioglitazone, take into account the most up-to-date advice from

regulatory authorities, cost, safety and prescribing issues.

**Exenatide**

● Continue exenatide only if the person has a reduction in HbA1c of ≥ 1.0 percentage point

and ≥ 3% of initial body weight in 6 months.

● Discuss the benefits of exenatide to allow the person to make an informed decision.

**Acarbose**

● Consider acarbose for a person unable to use other oral glucose-lowering medications.

**Starting insulin therapy**

● If other measures do not keep HbA1c to < 7.5% (or other agreed target), discuss benefits and

risks of insulin treatment.

● Initiate with a structured programme.

● Begin with human NPH insulin taken at bedtime or twice daily according to need.

● Alternatively, consider a once-daily long-acting insulin analogue (insulin detemir, insulin

glargine) if:

– the person needs help with injecting insulin and a long-acting insulin analogue would

reduce injections from twice to once daily, or

– the person’s lifestyle is restricted by recurrent symptomatic hypoglycaemic episodes, or

– the person would otherwise need twice-daily basal insulin injections plus oral glucose lowering

drugs, or

– the person cannot use the device to inject NPH insulin.

● Consider twice-daily biphasic human insulin (pre-mixed) (particularly if HbA1c ≥ 9.0%).

A once-daily regimen may be an option.

● Consider pre-mixed preparations of insulin analogues (including short-acting insulin

analogues) rather than pre-mixed human insulin preparations if:

– immediate injection before a meal is preferred, or

– hypoglycaemia is a problem, or

– blood glucose levels rise markedly after meals.

● Consider switching to a long-acting insulin analogue (insulin detemir, insulin glargine) from

NPH insulin if the person:

– does not reach target HbA1c because of hypoglycaemia, or

– has significant hypoglycaemia with NPH insulin irrespective of HbA1c level, or

– cannot use the delivery device for NPH insulin but could administer a long-acting

insulin analogue, or

– needs help to inject insulin and could reduce the number of injections with a long-acting

analogue.

● Review use of sulfonylurea if hypoglycaemia occurs with insulin plus sulfonylurea.

**Intensifying the insulin regimen**

● Monitor those using basal insulin regimens (NPH or a long-acting analogue [insulin detemir,

insulin glargine]) for need for short-acting insulin before meals or pre-mixed insulin.

● Monitor those using pre-mixed insulin once or twice daily for need for further injection of

short-acting insulin before meals or change to mealtime plus basal regimen.

**Insulin delivery devices**

● Offer education to a person who requires insulin about using an injection device (usually a

pen injector and cartridge or a disposable pen) that they and/or their carer find easy to use.

● If a person has a manual or visual disability and requires insulin, offer an appropriate device or

adaptation that can be used successfully.

● Appropriate local arrangements should be in place for the disposal of sharps.
