# Supplementary material for: Supported Telemonitoring and Glycemic Control in People with Type 2 Diabetes: The Telescot Diabetes Pragmatic Multicenter Randomized Controlled Trial
Source: PLoS Med. 2016 Jul 26;13(7):e1002098. doi: 10.1371/journal.pmed.1002098 (PMC4961438; doi:10.1371/journal.pmed.1002098)
Supplement: S5 Text — (DOCX) [file pmed.1002098.s015.docx]

**S5 Text: subgroup analysis for the Telescot diabetes pragmatic randomized controlled trial**

For the primary outcome, subgroup analyses were performed based on age, sex, socio-economic status of patients located in Scotland (based on the Scottish Index of Multiple Deprivation derived from postcode), baseline HbA1c, monitored systolic blood pressure and body mass index (BMI). These variables were categorised into tertiles and entered the linear regression model as two dummy variables representing the top two levels of the factor (except sex which remained binary). Subgroup analyses were conducted by adding interactions between these dummy variables and trial arm into the linear regression model and observing their significance. The main interest was in whether or not the effect of the telemonitoring intervention differed significantly across the different subgroups. Separate models were fitted for each subgroup variable, and all main effects terms were entered in the model with their corresponding interaction terms. Baseline HbA1c and the minimisation stratifiers were included as additional covariates if they were not already present in the models. The full results of the parameter estimates are given below for the outcome HbA1c (mmol/mol), but ultimately of most interest are the p-values corresponding to the interaction terms as highlighted. All models were based on N=285 participants. **No significant interactions were found (see tables S5-S10)**, and this conclusion was unchanged when using HbA1c measured as %.
