# Supplementary material for: Correction: Supported Telemonitoring and Glycemic Control in People with Type 2 Diabetes: The Telescot Diabetes Pragmatic Multicenter Randomized Controlled Trial
Source: PLoS Med. 2016 Oct 19;13(10):e1002163. doi: 10.1371/journal.pmed.1002163 (PMC5070826; doi:10.1371/journal.pmed.1002163)
Supplement: S4 Table — This file includes supplementary data. (DOCX) [file pmed.1002163.s001.docx]

**S4 Table Adverse events occurring during the trial by randomisation group**

| **Supported tele-monitoring** | **Usual care** |
| --- | --- |
| 2 hypoglycaemic episodes  1 hypotensive episode  1 peripheral angioplasty  1 myocardial infarction  1 admission to hospital with heart failure  1 cerebral infarction with haemorrhagic transformation | 2 hypoglycaemic episodes  1 abscess in the presence of uncontrolled glycaemia  1 ischaemic stroke  1 coronary angioplasty  1 hospital admission with a urinary tract infection and hyperglycaemia |
